# Ecdysone regulates *Drosophila* wing disc size via a TORC1 dependent mechanism

**DOI:** 10.1038/s41467-021-26780-0

**Published:** 2021-11-18

**Authors:** Katrin Strassburger, Marilena Lutz, Sandra Müller, Aurelio A. Teleman

**Affiliations:** 1 German Cancer Research Center (DKFZ), 69120 Heidelberg, Germany; 2 Heidelberg University, 69120 Heidelberg, Germany; 3 CellNetworks - Cluster of Excellence, Heidelberg University, Heidelberg, Germany; 4 Present Address: Technische Universität Dresden, 01217 Dresden, Germany

**Keywords:** Cell signalling, Developmental biology

## Abstract

Most cells in a developing organ stop proliferating when the organ reaches a correct, final size. The underlying molecular mechanisms are not understood. We find that in *Drosophila* the hormone ecdysone controls wing disc size. To study how ecdysone affects wing size, we inhibit endogenous ecdysone synthesis and feed larvae exogenous ecdysone in a dose-controlled manner. For any given ecdysone dose, discs stop proliferating at a particular size, with higher doses enabling discs to reach larger sizes. Termination of proliferation coincides with a drop in TORC1, but not Dpp or Yki signaling. Reactivating TORC1 bypasses the termination of proliferation, indicating that TORC1 is a main downstream effector causing proliferation termination at the maximal ecdysone-dependent size. Experimental manipulation of Dpp or Yki signaling can bypass proliferation termination in hinge and notum regions, but not the pouch, suggesting that the mechanisms regulating proliferation termination may be distinct in different disc regions.

## Abstract (web summary)

During development, organs grow to achieve a size necessary for their function; how final organ size is sensed to trigger growth arrest, however, remains unclear. Here the authors show that Drosophila wing disc size is determined by the hormone ecdysone through a TORC1-dependent mechanism.

## Introduction

During animal development, organs grow until they reach a suitable final size, at which point most cells in that organ stop proliferating. The correct termination of organ and tissue growth is critical for yielding animals of appropriate dimensions and proportions, and for preventing uncontrolled cell proliferation as occurs in cancer^1^. Although we understand much about the biological mechanisms that control organ patterning, we know comparatively little about how organ size is controlled^2,3^.

This fundamental question in developmental biology has been studied mainly in *Drosophila* using the developing wing anlage, the wing disc, which is initially specified as a group of 30 cells that proliferate to yield an organ comprising 50,000 cells at the end of larval development^2,4^. At that point, growth in the animal essentially ceases due to a spike in ecdysone hormone, which causes animals to stop feeding, become pupae, and undergo metamorphosis^5–7^. Wing disc cells perform two final rounds of reductive cell divisions and exit the cell cycle roughly 24 h after pupa formation^8–10^.

As the wing disc grows during larval development, wing disc cells undergo two separable processes: they grow (increase in biomass) and they proliferate (advance through the cell cycle and divide). Cell cycle progression per se does not lead to mass increase, and hence disc growth, as can be seen by genetic manipulations of cell cycle components^11,12^. For the disc to increase in mass, the cells need to increase in mass— i.e., grow. However, a diploid cell can only increase in size within a limited range. Hence the 1000-fold increase in the size of the wing disc during larval development requires combined growth and proliferation of wing disc cells. The final size of the wing disc is set when cells stop growing and proliferating.

The molecular mechanisms controlling growth termination are only starting to be understood. Several mechanisms have been ruled out. Disc cells do not count cell divisions to determine when to stop proliferating. If large fractions of the disc are killed genetically or with x-rays, the remaining cells compensate by proliferating more than usual, yielding normally sized organs^13–15^. Furthermore, accelerating or decelerating cell cycle progression in the wing disc leads to a normally sized organ composed of either more, smaller cells or fewer, larger cells, respectively^11,12^. Likewise, a mechanistic model where cells measure time has been excluded by slowing down disc growth using ‘Minute’ mutations, and seeing that discs compensate by extending developmental time, thereby achieving a normal size^16,17^. These experiments have revealed that size sensing occurs at the level of compartments in the wing (e.g., the posterior versus anterior compartments)^17^, and that this occurs prior to pupation^17^.

The hormone ecdysone plays an important, yet dichotomous role, in regulating wing disc size. High levels of ecdysone which occur at the onset of metamorphosis cause differentiation of wing disc cells and their arrest in G2^10,18^. Hence this peak of ecdysone leads to termination of growth and proliferation in the wing. Indeed, a premature ecdysone peak causes premature termination of growth, yielding small animals^19^, whereas a delay in the ecdysone peak yields larger animals^20,21^. In contrast, the lower levels of ecdysone present during larval stages promote wing disc cell proliferation both in vivo^3,16,18,22,23^ and in cultured explants^24,25^. The downstream molecular mechanisms by which ecdysone influences wing cell proliferation are only partly understood^22^. Also unclear is whether ecdysone simply plays a permissive function in allowing wing cells to proliferate, or whether ecdysone levels control final wing disc size.

We study here how ecdysone affects *Drosophila* wing disc growth. To do so, we abolish endogenous ecdysone synthesis and reconstitute ecdysone signaling in vivo at different levels by feeding the bioactive form 20-hydroxy-ecdysone (20E) in a dose-controlled manner. We find that ecdysone supports wing disc growth in a dose-dependent manner, with higher levels of ecdysone enabling wing discs to reach a larger size. Consistent with this, the ability of ecdysone to support wing cell proliferation depends on the size of the wing disc: A fixed level of ecdysone signaling is able to promote proliferation of wing disc cells if they reside in a disc of small size, but not in a disc of larger size. We then study the downstream signaling pathways that cause wing discs to terminate proliferation for a given level of ecdysone signaling. We find that a drop in TORC1 signaling, but not yki or dpp signaling, causes the termination of cell proliferation, which is unexpected given that TORC1 mainly promotes cell growth and not cell proliferation^26,27^.

## Results

### Constant exogenous 20E allows wing disc proliferation

During *Drosophila* development, ecdysone titers display complex dynamics, spiking above baseline multiple times to promote developmental transitions (Supplementary Fig. 1a). To study the effect of ecdysone signaling on wing disc proliferation in a controlled manner, we aimed to abolish the endogenous synthesis of ecdysone and to replace it with exogenously fed ecdysone at fixed levels. To this end, we knocked-down *spookier* (*spok*), an enzyme in the ecdysone biosynthesis pathway^28,29^, using a temperature-sensitive inducible system (Tub-Gal4, Tub-Gal80^ts^, hereafter referred to as Tub^ts^ > *spok*^*i*^). We did this at the beginning of the third larval instar (L3), after the spike in ecdysone at the L2/L3 transition had enabled animals to complete their last larval molt (Supplementary Fig. 1a). Since *spok* is expressed only in the prothoracic gland where ecdysone is made, we used a ubiquitous tubulin-GAL4 driver to express *spok* RNAi (*spok*^*i*^), since this leaves wing discs unperturbed yet enables us in subsequent experiments to also knock down additional test genes in the wing disc. Alternatively, we obtained similar results with phm-GAL4 for prothoracic gland-specific knockdown of *spok*, or with a combined Gal4/LexA system (see below). We monitored ecdysone signaling in whole larvae by measuring mRNA levels of the low-threshold ecdysone target gene *Eip71CD* (also known as *Eip28*/*29*) that is induced by baseline ecdysone levels present in early L3 larvae^30^. This revealed that ecdysone signaling was reduced within 24 h of *spok* RNAi induction (Supplementary Fig. 1b). This inducible system allowed *spok*^*i*^ animals to reach third instar, yet efficiently prevented them from pupating (Supplementary Fig. 1c). Whereas control animals pupated 72 h after RNAi induction, and showed increased *Eip71CD* expression, *spok*^*i*^ animals did not (Supplementary Fig. 1d). Instead, *spok*^*i*^ animals continue feeding and remain active as larvae for an extended period of time. Roughly half of the animals survive as larvae for 2 weeks (Supplementary Fig. 1e), significantly extending the third instar larval period which usually lasts 2 days.

We next asked what happens to wing disc proliferation in Tub^ts^ > *spok*^*i*^ animals, using EdU incorporation as a readout for progression through S-phase. Although wing discs continued proliferating for the first 12 h after RNAi induction, they subsequently progressively become EdU negative at 24 and 48 h after RNAi induction despite still being small (Supplementary Fig. 1f, quantified in Supplementary Fig. 1f’). This is consistent with our previous findings using tissue explants^25^, as well findings from other labs^16,22^, that some ecdysone signaling is required for wing discs to proliferate. Since *spok* RNAi is reducing ecdysone signaling below the levels of normal L3 larvae (Supplementary Fig. 1b), this is not sufficient to maintain wing discs in a proliferative state.

To provide wing discs a constant, low-level supply of ecdysone, we supplemented the food of *spok*^*i*^ animals with the active form of the hormone, 20-hydroxyecdysone (20E). We titrated 3–400 µM 20E into the food and found that 25 µM 20E was sufficient to restore ecdysone signaling to the level of similarly aged control larvae (Supplementary Fig. 2a–b). Restoring ecdysone signaling also enabled wing discs to stay proliferative 48 h after *spok* RNAi induction (Supplementary Fig. 2c–d, quantified in Supplementary Fig. 2e–f). In addition to cell cycle progression, 25 µM 20E also visibly promoted tissue growth (compare disc size in 0 µM vs 25 µM Supplementary Fig. 2c, quantified in more detail below). Similar results were obtained by knocking down *spok* specifically in the prothoracic gland with phm-GAL4 (Supplementary Fig. 3).

### Discs with constant 20E stop proliferating at the right size

**Fig. 1:**
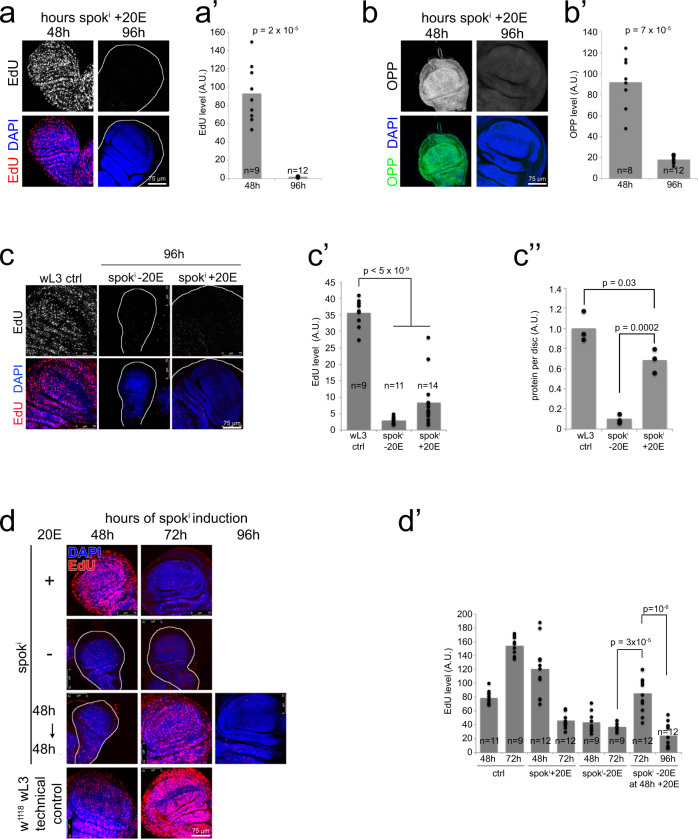
Exogenously controlled ecdysone signaling enables wing discs to first proliferate and then terminate growth and proliferation. **a**–**b**’ Tub^ts^ > *spok*^*i*^ + 20E discs first proliferate (**a**–**a**’) and grow (**b**–**b**’) robustly and then stop. **a**–**a**’ Proliferation, assayed by EdU incorporation, for *spok*^*i*^ discs 48 or 96 h after knockdown induction and 20E feeding. Representative images in (**a**), quantified in (**a**’). Representative of six biological replicates. **b**–**b**’ Growth, assayed via O-propargyl-puromycin (OPP) incorporation into nascent polypeptide chains, 48 or 96 h after knockdown induction and 20E feeding. Representative images in (**b**), quantified in (**b**’) Representative of three biological replicates. **c**–**c**” Tub^ts^ > *spok*^*i*^ + 20E discs grow 10-fold in size and then terminate proliferation at a size similar to those of control wing discs at the end of third instar larval development. Proliferation is assayed by EdU incorporation, representative images in (**c**), quantified in (**c**’) and size is assayed as total protein per disc by BCA protein measurement (**c**”, *n* = 3 × 2 technical duplicates on 6 wing discs). **d**–**d**’ Discs proliferate beyond the 48 h timepoint if 20E feeding is delayed. EdU incorporation at indicated times after *spok*^*i*^ induction for larvae not fed ecdysone (−20E), fed ecdysone as of knockdown induction (+20E) or fed ecdysone starting 48 h after knockdown induction (48 h− → 48 h+). The w^1118^ technical control is not grown under the same conditions as the other samples, and only serves to confirm that the EdU staining worked. Representative images in (**d**), quantified in (**d**’). Representative of two biological replicates. For all quantifications *p*-values determined by *t*-test.

Given that Tub^ts^ > *spok*^*i*^ + 20E wing discs are hosted in a larval environment for many days with constant exogenous ecdysone levels, we asked whether the wing discs eventually stop growing and proliferating, or whether they grow and proliferate indefinitely. We assayed growth via incorporation of O-propargyl-puromycin (OPP), which labels nascent polypeptides and is a quantitative readout for translation rates in cells. At 48 h after temperature shift in the presence of 25 µM 20E, wing discs display incorporation of both EdU and OPP, indicating that they are actively proliferating and growing (Fig. 1a, b). Interestingly, by 96 h wing disc cells stop proliferating and growing (Fig. 1a, b, quantified in Fig. 1a’, b’). During this time, the wing discs have increased in size to nearly the size of wild-type wL3 discs (Fig. 1c–c”). Two possible explanations for the termination of growth and proliferation are: (1) the larval environment is not capable of promoting growth and proliferation for more than 48 h or (2) wing discs terminate proliferation because they have reached their final size. To test this, we delayed ecdysone feeding and started it only at 48 h after RNAi induction, in order to delay wing disc growth, while larval tissues continue growing. As expected, wing discs of animals where we knockdown *spok* and immediately feed ecdysone are strongly proliferative at 48 h after RNAi induction and have terminated proliferation 72 h after RNAi induction (Fig. 1d–d’, “+20E” samples). Also as expected, wing discs in *spok*^*i*^ animals that are not fed ecdysone are small and non-proliferative at both 48 h and 72 h after RNAi induction (Fig. 1d–d’, “−20E”). If we take some of these animals and start feeding them ecdysone at 48 h, when the discs are still small and EdU negative, they recommence proliferation and growth, and now are strongly EdU positive at 72 h, and then terminate proliferation at 96 h (Fig. 1d–d’, “48 h− → 48 h+” samples). This suggests that larvae after 48 h are still competent to support wing disc proliferation. As additional evidence that these larvae are competent to support wing disc growth and proliferation after 48 h, as presented below, wing discs mutant for *wts* or TSC2 can sustain proliferation beyond these timepoints, and achieve larger final sizes. Furthermore, wing discs that have stopped proliferating do not display cell death (Supplementary Fig. 4). In sum, wing discs in the Tub^ts^ > *spok*^*i*^ + 20E system stop growing and proliferating at roughly the right size, despite the lack of an ecdysone pulse that normally stops growth and proliferation extrinsically. This is in line with recent findings from the Struhl lab, published while our manuscript was in revision, using a similar system^31^.

### 20E promotes proliferation depending on disc size

**Fig. 2:**
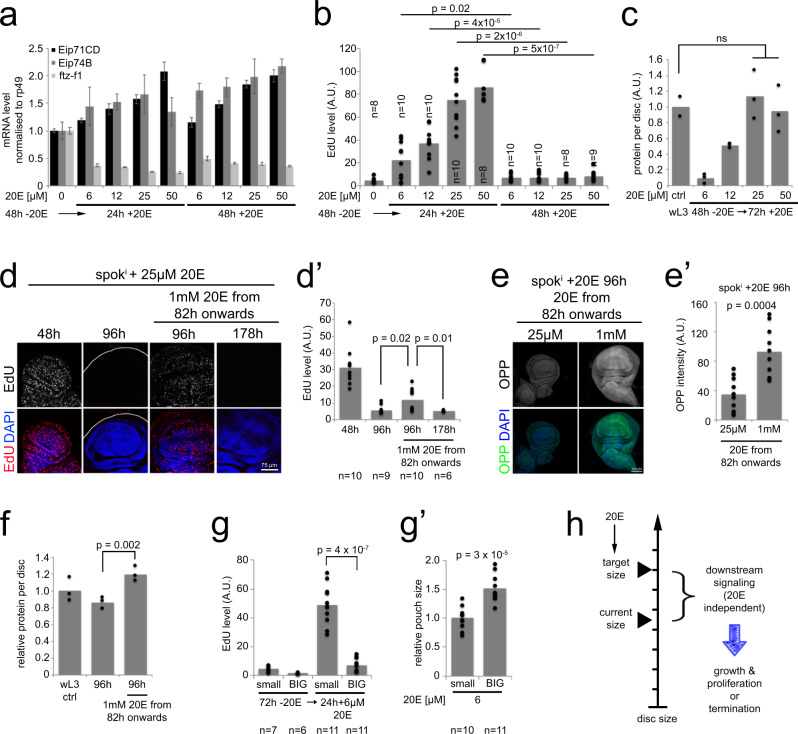
Ecdysone signaling does not decrease when wing discs stop proliferating, but it affects the target size. **a**–**b** Ecdysone signaling does not change when Tub^ts^ > *spok*^*i*^ + 20E discs stop proliferating. Knockdown of *spok*^*i*^ (Tub^ts^ > *spok*^*i*^) was induced 48 h prior to feeding larvae with 6–50 µM 20E in order to start with a low baseline. **a** Ecdysone target gene expression was measured by Q-RT-PCR 24 h and 48 h after 20E feeding, whereby Eip71C and Eip74B are induced by 20E and ftz-f1 is repressed by 20E. *n* = 10 discs/condition. Representative of two biological replicates. Bar = mean. Error bars = std. dev. **b** For all ecdysone signaling levels, Tub^ts^ > *spok*^*i*^ + 20E discs stop proliferating by 48 h after 20E supplementation, measured by EdU incorporation. Representative of two biological replicates. **c**–**f** Final disc size depends on ecdysone concentration. **c** A titration of 6–50 µM 20E was fed to Tub^ts^ > *spok*^*i*^ larvae as in (**a**–**b**). Disc size is assayed as total protein per disc by BCA protein measurement 72 h after 20E feeding. *n* = 6 discs/sample. Representative of two biological replicates. **d**–**f** Wing discs from Tub^ts^ > *spok*^*i*^ + 20E larvae raised on 25 µM 20E can be induced to proliferate (**d**–**d**’) and grow (**e**–**e**’) further by feeding a higher 20E concentration (1 mM), leading to larger wing discs (total protein per disc, **f**). Representative pictures of EdU or OPP incorporation (**d** and **e**) and quantifications (d’ and **e**’) are shown. *n* = 10 discs/condition (**e**–**e**’) or 6 discs/condition (**f**) × 2 biological replicates. **g**–**g**’ The same concentration of ecdysone stimulates proliferation more strongly in small discs than big discs. Knockdown of *spok*^*i*^ was induced for 72 h. By visual inspection Tub^ts^ > *spok*^*i*^ larvae were separated into “small” and “BIG” prior to 6 µM 20E feeding. Proliferation, measured by EdU incorporation (**g**) and pouch size (**g**’) were determined 24 h after 20E supplementation. Representative of two biological replicates. **h** A given concentration of ecdysone enables wing discs to grow only up to a certain maximal ‘target’ size. When wing discs reach that size, they stop proliferating and growing, although ecdysone signaling does not turn off. For all quantifications *p*-values determined by *t*-test.

We next used this *spok*^*i*^ system to test whether exogenous ecdysone promotes disc growth in a concentration-dependent manner. To this end, we first starved Tub^ts^ > *spok*^*i*^ animals of ecdysone for 48 h, causing ecdysone signaling to drop (Supplementary Fig. 1b) and wing discs to stop proliferating (Supplementary Fig. 1f–f’), and then added 20E to the food. This assures that the signaling and proliferation we subsequently observe are due to the exogenously supplied ecdysone. We titrated 20E in the food from 6 to 50 µM. This led to a dose-dependent induction of 20E signaling in the wing disc, assayed by Q-RT-PCR of *Eip71CD* and *Eip74B*, which are induced by ecdysone signaling, and *ftz-f1* which is inhibited by ecdysone (Fig. 2a). Induction of ecdysone signaling was stable across time, seen by comparing 24 h versus 48 h after feeding (Fig. 2a). Increasing 20E levels led to a dose-dependent increase in EdU incorporation in the wing disc at 24 h (Fig. 2b). This can either be via an autonomous, direct action of ecdysone on the wing disc, or via a signaling relay with ecdysone acting on another organ. In all 20E concentration conditions, however, wing discs terminated proliferation by 48 h (Fig. 2b). (Note that in this setup, wing discs proliferate somewhat during the first 48 h as ecdysone titers drop, thereby requiring only 48 h of exogenous ecdysone to reach final size.) Hence all the concentrations of 20E we tested were sufficient to support wing disc proliferation, but none was capable of maintaining wing disc proliferation indefinitely (see also 1 mM 20E, Fig. 2d below). Notably, 20E signaling did not drop in wing discs that had terminated proliferation at 48 h compared to 24 h (Fig. 2a). Hence wing disc cells do not stop proliferating due to a change in ecdysone signaling. This is conceptually different from situations where ecdysone signaling is experimentally reduced, thereby causing a proliferation block^22^. Since wing discs stop proliferating without a decrease in ecdysone signaling, some other signal must become limiting to cause the proliferation stop.

We next tested if the final size of the wing disc depends on 20E concentration. Indeed, the more ecdysone the animals were fed, the bigger the discs grew, with 25 µM or 50 µM 20E enabling wing discs to reach the expected final disc size of wandering L3 animals (Fig. 2c). Hence, 20E affects final disc size. We found this to be an unexpected and interesting result because it has a number of conceptual implications. Firstly, it suggests that the larger a wing disc gets, the more ecdysone signaling it needs to continue growing: for instance, for wing discs up to the size of 0.5 in Fig. 2c, 12 µM 20E is sufficient for the disc to continue growing. Above this size, however, wing discs need 25 µM 20E to continue growing. A second implication, which is the flip-side of the first, is that the ability of ecdysone to promote proliferation of a wing disc cell depends on the size of the wing disc in which the cell resides: a fixed level of ecdysone signaling (e.g., 12 µM in Fig. 2c) can support growth and proliferation of wing disc cells when the disc is below a certain size (0.5 in Fig. 2c) but not when the wing disc is larger. We decided to test these two implications directly. We tested the first implication by feeding animals 25 µM 20E and then increasing the ecdysone dose to 1 mM. With only 25 µM 20E, wing discs terminate proliferation at 96 h (Fig. 2d). If instead at 82 h we increase the 20E dose 1 mM, this induced the wing discs to transiently grow and proliferate further (Fig. 2d–e’), reaching a larger size (Fig. 2f). We then tested the second implication—that a given concentration of ecdysone causes cells to proliferate when they are part of a small wing disc, but fails to promote their proliferation if the cells are part of a wing disc above a size threshold. To test this in a controlled manner, we grew Tub^ts^ > *spok*^*i*^ animals for 72 h in the absence of 20E to inactivate endogenous ecdysone signaling, leading to a block in cell proliferation (Fig. 2g). Larvae were then separated into two size groups by visual inspection (Fig. 2g’), fed 6 µM 20E, and inspected 24 h later for cell proliferation. This confirmed that 6 µM 20E induced cell proliferation in small wing discs but hardly in large wing discs (Fig. 2g).

Altogether, these data are consistent with a model whereby ecdysone does not directly drive wing cell proliferation, otherwise it would do so equally to cells in small or big discs. Instead, each concentration of ecdysone can sustain disc growth up to a certain target size (Fig. 2h). Cells in discs below this ecdysone-dependent target size are capable to proliferate, but not cells above this target size. When wing discs reach this ecdysone-dependent target size, this causes some other downstream signal to become limiting for cell proliferation because ecdysone signaling in disc cells does not change as the disc reaches this target size (Fig. 2a–b, h). These data suggest that as larvae approach the end of 3^rd^ instar, where ecdysone levels progressively increase^30^, they progressively adjust upwards their target size. This is consistent with previous work showing that damage to one disc causes the other discs to slow proliferation via reduced ecdysone signaling^16^.

### Dpp and Hpo/Yki signaling when discs stop proliferating

**Fig. 3:**
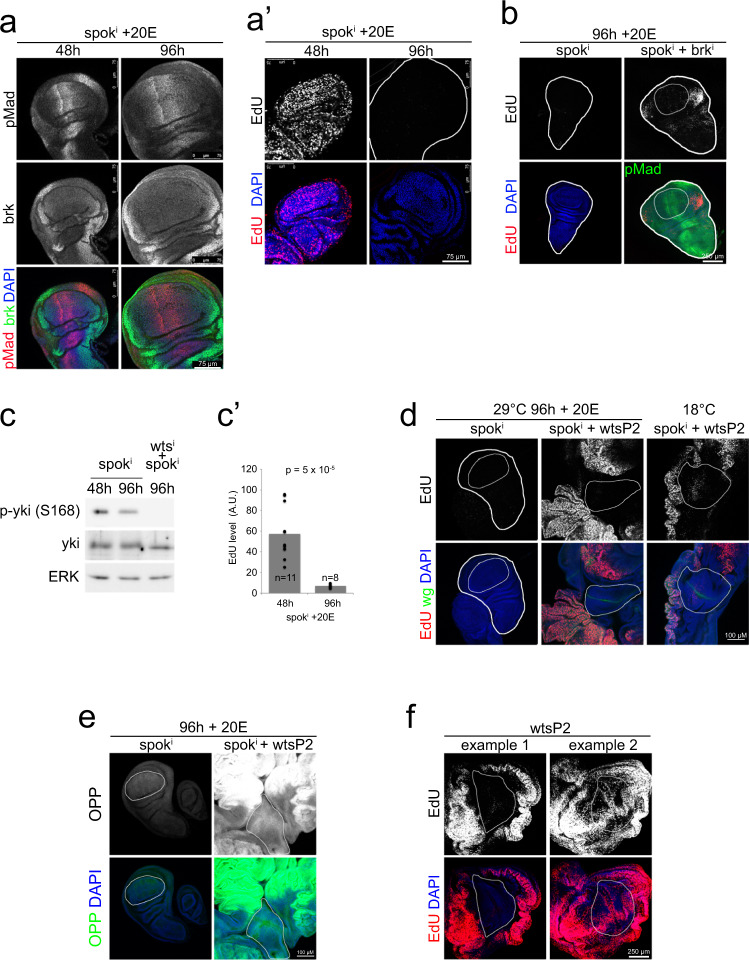
For a given ecdysone concentration, proliferation in the wing pouch terminates successfully in the absence of *warts* or *brinker*. **a**–**a**’ Dpp signaling activity, assayed by immunostaining Tub^ts^ > *spok*^*i*^ + 20E discs with pMad and brk antibodies (**a**), is similar in proliferating discs and in discs that have terminated proliferation (**a**’), 48 and 96 h after knockdown induction, respectively. Proliferation was assayed by EdU incorporation. *n* = 10 discs/condition × 3 biological replicates. **b** The wing pouch terminates proliferation also upon knockdown of *brk*. 96 h after knockdown induction, Tub^ts^ > *spok*^*i*^ + 20E discs have terminated proliferation, assayed by EdU incorporation. Most regions of discs with concomitant knockdown of *brk* (*spok*^*i*^ + *brk*^*i*^) also stop proliferating with only the very lateral regions bypassing the stop. Thick white line outlines the disc, thin white line outlines the pouch. *n* = 10 discs/condition × 3 biological replicates. **c**–**c**’ Global yki activity does not drop in Tub^ts^ > *spok*^*i*^ + 20E discs when they terminate proliferation. **c** Phosphorylation of yki measured by immunoblotting lysates of wing discs 48 h or 96 h after knockdown induction from Tub^ts^ > *spok*^*i*^ + 20E or Tub^ts^ > *spok*^*i*^ + *wts*^i^ + 20E animals. n = 40 discs/condition × 3 biological replicates. **c**’ Proliferation was measured as a control via EdU incorporation. Representative of three biological replicates. *P*-value determined by *t*-test. **d** *Wts* loss of function in Tub^ts^ > *spok*^*i*^ + 20E discs (*spok*^*i*^ + *wts*^*P2*^) leads to overproliferation in proximal regions, assayed by EdU incorporation, whereas the pouch (white outline), identified by a *wg* expression stripe, terminates proliferation 96 h after knockdown induction and 20E feeding (29 °C 96 h + 20E). Without *spok*^*i*^ knockdown (18 °C *spok*^*i*^ + *wts*^*P2*^) *wts*^*P2*^ discs also stop proliferating in the pouch (white outline) with overproliferating proximal regions. *n* = 10 discs/condition × 3 biological replicates. **e** The wing pouch (white outline) of *spok*^*i*^ + *wts*^*P2*^ discs has reduced growth compared to proximal regions, assayed by OPP incorporation, 96 h after knockdown induction and 20E feeding. *n* = 6 discs. **f** The wing pouch of *wts*^*P2*^ (in WT background) discs can stop proliferating in L3 larvae. A range of phenotypes is observed, from example 1, where the pouch is completely EdU negative, to example 2, where the compartment boundaries are still EdU positive and the quadrants are EdU negative. *n* = 10 discs/condition × 3 biological replicates.

We next asked what downstream signaling pathways are responsible for terminating the proliferation of wing discs when they reach the ecdysone-dependent target size. For this purpose, we selected 25 µM 20E as a fixed ecdysone concentration, since it supports wing disc growth up to normal wandering L3 size (Fig. 2c), and studied which signaling pathways turn off when the wing discs stop proliferating. We first assayed whether Dpp signaling turns off when proliferation stops. We stained wing discs for phospho-Mad, which reads-out Dpp signaling activity^32^, and for brk, which is expressed where Dpp signaling is low, and acts to repress proliferation. This revealed that Dpp signaling is very similar in wing discs 48 h after *spok* RNAi induction, when they are small and proliferative, and 96 h after *spok* RNAi induction, when growth and proliferation have terminated (Fig. 3a–a’). In both cases, Dpp signaling is high medially, as seen by the high pMad levels, and low laterally where brk is expressed. Hence Dpp signaling does not appear to turn off at the time when proliferation stops. We next tested functionally whether the Dpp/Brk pathway plays a role in the termination of proliferation. To this end, we knocked down *brk* by RNAi in the wing disc, since brk mediates the effects of Dpp signaling on cell proliferation^33,34^. Wing discs lacking *brk* still terminate proliferation in most regions of the disc, including all of the wing pouch which will form the adult wing blade (Fig. 3b, Supplementary Fig. 5a). Hence brk is not required for termination of proliferation in most regions of the wing disc. In the absence of *brk*, however, wing discs develop lateral bulges, and some wing discs, but not all, show some cell proliferation in these regions at 96 h (Fig. 3b, Supplementary Fig. 5a). Similar to the Dpp pathway, the wingless pathway also promotes wing disc growth and proliferation^35^. The wing pouch of wing discs expressing arm^S10^, which constitutively activates wingless signaling, still terminates proliferating at 96 h (Supplementary Fig. 5b). In sum, although Dpp and wingless signaling can modulate proliferation rates in the wing, neither determines the termination of proliferation.

We next addressed the role of Hippo/Yorkie (Hpo/Yki) signaling in growth termination. We first tested if yki activity drops when wing discs stop proliferating, by quantifying yki phosphorylation with immunoblots of wing disc lysates. Since phosphorylation inactivates yki, we would expect phospho-yki levels to increase when proliferation stops. However, phospho-yki levels did not increase in wing discs 96 h after *spok* RNAi induction (Fig. 3c) when the wing discs were no longer proliferative (Fig. 3c’). We also quantified the subcellular localization of yki in the wing pouch, since the cytosolic-to-nuclear ratio of yki reflects its activity. As previously shown^36^ yki becomes less nuclear in wing disc pouches of wild-type larvae as they reach the end of L3 (Supplementary Fig. 5c). In the Tub^ts^ > *spok*^*i*^ + 20E system, we did not observe relocalization of yki to the cytoplasm in the pouch when wing discs terminate proliferation (Supplementary Fig. 5c), in agreement with our immunoblots (Fig. 3c). Furthermore, the amount of nuclear yki was within the range of nuclear yki seen in mid-L3 and wandering L3 wild-type discs, both of which are proliferative (Supplementary Fig. 5c). Nonetheless, we tested functionally whether constitutive activation of yki via knockdown of *warts* (*wts*) can bypass the termination of proliferation. Surprisingly, *wts* knockdown has different effects in different regions of the wing disc. The wing pouch, which we identified morphologically (white outline, Supplementary Fig. 5d), and via the characteristic ZNP double-stripe which is visible by DAPI staining (arrow, Supplementary Fig. 5d), and by a stripe of *wingless* (*wg*) expression at the D/V boundary, still terminates proliferation in the absence of *wts* (Supplementary Fig. 5d, “example 1”). This result was unexpected because it indicates that the wing pouch stops proliferating despite active yki. The lateral regions along the D/V boundary—the ones that remain proliferative in the *brk* knockdown discs (Fig. 3b)—also become EdU negative in the absence of *wts* (Supplementary Fig. 5d). However, both the dorsal and the ventral regions outside the pouch and further away from the D/V boundary remain strongly proliferative in the absence *wts*, and massively overgrow leading to tumor-like masses (example 2, Supplementary Fig. 5d). OPP incorporation is also lower in the pouch compared to surrounding tissue (Supplementary Fig. 5e), indicating reduced growth concomitant with the termination of proliferation, and consistent with the size of the wing pouch remaining roughly normal. As a control, we confirmed that wing discs with *wts* knockdown display hallmarks for activated yki: low yki phosphorylation (Fig. 3c) and nuclear yki localization (Supplementary Fig. 5f). The same results for growth and proliferation were obtained by introducing the wing-disc-specific wts^P2^ mutation, instead of *wts*-RNAi, into the *spok*^*i*^ background (29 °C in Fig. 3d, e). In sum, dpp signaling, wingless signaling, and yorkie signaling do not dictate the termination of growth and proliferation in the wing disc pouch when it reaches the ecdysone-dependent target size, because signaling through these pathways does not decrease at the timepoint of termination, and because wing pouches terminate growth and proliferation despite experimentally hyperactivated dpp, wingless and yorkie signaling.

We asked whether similar results can be observed in animals where we do not experimentally control ecdysone signaling. We first analyzed wing discs from Tub^ts^ > *spok*^*i*^ + wts^P2^ larvae that were not shifted to 29 °C, thereby allowing unperturbed endogenous 20E synthesis, and found that also in these discs the pouch terminates proliferating at roughly the correct size while proximal areas continue proliferating, leading to massive overgrowths (18 °C, Fig. 3d). Similarly, wing discs from wts^P2^ L3 larvae in an otherwise wild-type background show a range of phenotypes, with some wing pouches completely EdU-negative and some wing pouches containing proliferative cells along the compartment boundaries with EdU-negative quadrants (Fig. 3f). Hence also in animals were ecdysone signaling is not experimentally controlled, the wing pouch can terminate proliferation despite hyperactive yki.

### Low TORC1 stops proliferation at the 20E-dependent target size

**Fig. 4:**
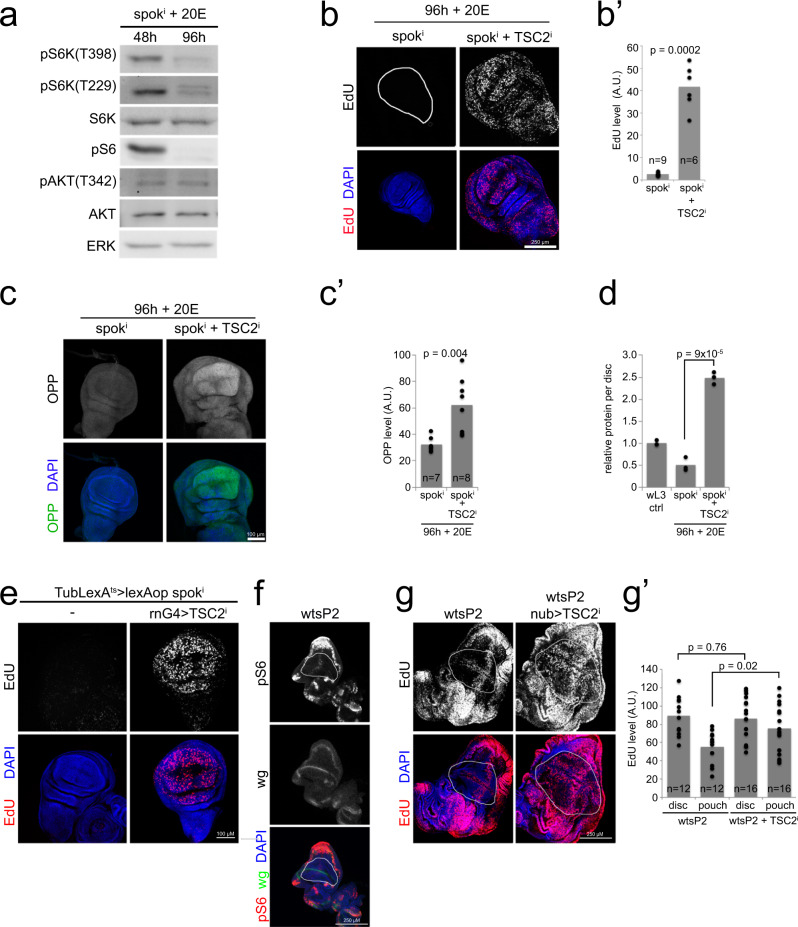
When a wing disc reaches the maximal size supported by a given concentration of ecdysone, the wing pouch terminates proliferation due to low TORC1 activity. **a** TORC1 activity drops when Tub^ts^ > *spok*^*i*^ + 20E discs terminate proliferation at 96 h. Phosphorylation of S6K on Thr398 is a direct readout for TORC1 activity. Phosphorylation on Thr229 by PDK1 requires a priming phosphorylation on Thr398. *n* = 40 discs/condition × 3 biological replicates. **b**–**d** Constitutive activation of TORC1 overcomes the termination of proliferation (**b**–**b**’) and growth (**c**–**c**’) in Tub^ts^ > *spok*^*i*^ + 20E discs, causing them to overgrow greatly (**d**). While Tub^ts^ > *spok*^*i*^ + 20E discs (*spok*^*i*^ 96 h) have terminated proliferation, judged by EdU incorporation, discs with concomitant knockdown of *TSC2* (*spok*^*i*^ + *TSC2*^*i*^ 96 h) continue proliferating. Representative images (**b** and **c**), quantified in (**b**’ and **c**’). Representative of 4 (**b**–**b**’) or 3 (**c**–**c**’) biological replicates. **d** Disc size at 96 h, assayed as total protein per disc by BCA. *n* = 6 discs/condition. Representative of three biological replicates. **e** Wing-pouch-specific knockdown of TSC2 bypasses the termination of proliferation in spok^i^ + 20E animals. Spok^i^ is expressed using the lexA/lexAop system combined with Gal80^ts^, and UAS-TSC2^i^ is expressed specifically in the wing pouch with rotund-GAL4 (rnG4). As expected, wing discs stop proliferating in spok^i^ + 20E animals at 96 h, and this is bypassed by rnG4 > TSC2^i^. *n* = 13 discs. **f** TORC1 activity drops in the pouch (white outline) of wts^P2^ L3 larvae, measured by immunostaining discs with phospho-S6 antibody. Representative of two replicates. **g**–**g**’ Activation of TORC1 in the pouch (white outline) with nubG4 in wts^P2^ L3 wing discs (wts^P2^, nub>TSC2^i^) overcomes the stop of proliferation in the pouch. Representative images (**g**), quantified in (**g**’). Representative of two replicates. For all quantifications *p*-values determined by *t*-test.

The results presented above indicate that cells in the wing pouch, which gives rise to the wing blade, terminate proliferation without a drop in dpp, wingless, yorkie or ecdysone signaling. Hence something else that is required for proliferation is likely becoming limiting. One regulator of cell growth is TORC1^26,37,38^. Although TORC1 does not directly regulate cell cycle progression, TORC1 activity is required for cell growth, which in turn is necessary for cell cycle progression^39^. Immunoblots of lysates from wing discs that had terminated proliferation showed a drop in S6K phosphorylation on Thr398, a direct readout for TORC1 activity (Fig. 4a, Supplementary Fig. 6a). As a consequence, also phosphorylation of S6K on Thr229, the PDK1 site which requires phosphorylation on Thr398 as a priming site, as well as phosphorylation of RpS6 was reduced (Fig. 4a). Phosphorylation of S6K on Thr229 was detected with an antibody that detects the PDK1 phosphorylation sites on both Akt and S6K (Supplementary Fig. 6b and ref. ^40^). The drop in TORC1 activity was similar in magnitude to the one obtained by feeding animals rapamycin (Supplementary Fig. 6c). Consistent with a drop in TORC1 activity, autophagy was mildly induced in ‘stopped’ wing discs (Supplementary Fig. 6d–d’). Hence, when discs reach the ecdysone-dependent target size, termination of growth and proliferation coincides with a drop in TORC1 activity.

To determine whether the decrease in TORC1 activity is upstream or downstream of the termination of cell proliferation, we genetically activated TORC1. If TORC1 inactivation is downstream of a cell cycle block, then re-activating TORC1 should not affect cell proliferation. If, instead, TORC1 inactivation is upstream and causing the cell cycle block, then genetically activating TORC1 should enable wing disc cells to continue proliferating. Activation of TORC1 via knockdown of *TSC2* (Supplementary Fig. 6a) caused cells in the wing pouch, as well as the rest of the disc, to continue proliferating and growing at 96 h when control wing discs stop (Fig. 4b–c’), indicating that the inactivation of TORC1 is upstream of the cell cycle block. *TSC2* knockdown discs become more than twice as large as control discs by 96 h (Fig. 4d) and were still proliferating at the latest timepoint we checked, 120 h (Supplementary Fig. 7a–a’). This phenotype was recapitulated with several *TSC2* RNAi lines: a VDRC KK line where we recombined away the second insertion at cytological location 40D (Supplementary Fig. 7b–b’) as well as an independent line from the TRiP collection (Supplementary Fig. 7c–c’). We confirmed in 3 different ways that this was due to a tissue-autonomous effect of *TSC2* knockdown in the wing disc: Knockdown of *TSC2* in imaginal discs and the prothoracic gland, using a combination of Ubx-Flp^41^ and phmG4, also prevented the termination of proliferation (Supplementary Fig. 8a–a’) whereas knockdown of *TSC2* only in the prothoracic gland did not (Supplementary Fig. 8b–b’). Flip-out clones in the wing disc expressing *TSC2* RNAi also did not terminate proliferation at 96 h (Supplementary Fig. 8c). Finally, we expressed *spok*^*i*^ using the lexA/lexAop system and specifically knocked down *TSC2* in the wing pouch with rotund-GAL4, and this also led to a bypass of proliferation termination in the wing (Fig. 4e). Since we previously showed that the cell cycle promotes TORC1 via CycD/Cdk4 in the wing disc^42^, we also tested the option that TORC1 is downstream of a cell cycle block by overexpressing CycD/Cdk4, however, this did not cause cells to continue proliferating (Supplementary Fig. 8d). These data indicate that TORC1 is downstream of the ecdysone-dependent set-point, and is mediating the proliferative response. Indeed, increasing the concentration of 20E fed to animals from 25 µM to 1 mM the last 14 h prior to dissection led to an increase in TORC1 signaling (Supplementary Fig. 9a), showing that it is downstream of, and activated by, ecdysone. Furthermore, knockdown of *TSC2* either ubiquitously or specifically in the wing pouch rescued proliferation in the absence of ecdysone in Tub^ts^ > *spok*^*i*^ animals fed no ecdysone (Supplementary Fig. 9b–c), showing epistatically that TORC1 is one of the main effectors regulating cell proliferation downstream of ecdysone signaling. In sum, low TORC1 activity is the limiting downstream factor causing wing discs to terminate growth and proliferation when they reach the ecdysone-dependent target size (Supplementary Fig. 9d).

We asked whether TORC1 activity also becomes limiting for pouch proliferation in a system where we do not experimentally control ecdysone signaling. In wts^P2^ mutant discs where the pouch stops proliferating (Fig. 3f), TORC1 activity is also low in the pouch (Fig. 4f). Knockdown of *TSC2* specifically in the pouch of wts^P2^ wing discs significantly increases EdU incorporation in the pouch (Fig. 4g–g’) whereas knockdown of *brinker* does not (Supplementary Fig. 9d). We also assayed TORC1 activity in wing discs of wild-type pre-wandering and wandering L3 larvae by immunoblotting for pS6 and pS6K (Supplementary Fig. 9f) and by quantifying levels of an unk-GFP reporter which we previously showed to be repressed by TORC1^43^ (Supplementary Fig. 9g) and found that TORC1 activity is reduced in wL3 compared to pre-wandering larvae. This is consistent with a slow-down but not complete block of cell proliferation in wL3 larvae^4^.

### Akt and Erk activity when discs stop proliferating

We next asked what is down-regulating TORC1 activity when wing discs reach the ecdysone-dependent target size. Although Akt is one upstream regulator of TORC1, in *Drosophila* we previously showed that Akt only regulates TORC1 in the ovary, and not other tissues^44^. Nonetheless, we assayed Akt activity in multiple different ways and found that it does not drop when wing discs terminate growth and proliferation. Phosphorylation of Akt on the PDK1 and TORC2 sites does not decrease (Fig. 4a, Supplementary Fig. 10a), indicating that growth-factor signaling via PI3K is not changing when discs terminate proliferation. If anything, phosphorylation of Akt increases, consistent with a negative feedback loop from TORC1 to PI3K and TORC2. Blotting for all Akt substrates with a pan Akt phospho-substrate antibody on wing disc lysates did not show a drop at 96 h (Supplementary Fig. 10b). Phosphorylation of GSK3b on Ser9, a direct readout for Akt activity, did not drop on a western blot (Supplementary Fig. 10c) nor by wing disc immunostaining (Supplementary Fig. 10d–e). Finally, the cytosolic localization of FOXO, which requires Akt activity, also did not change (Supplementary Fig. 10f). Another upstream regulator of TORC1 is Erk, but Erk phosphorylation also did not change (Supplementary Fig. 10a). Nutrients signal to TORC1 via the Rag GTPases. However, expression of constitutively active RagC did not bypass the termination of proliferation (Supplementary Fig. 10g). Hence further work will be required to understand the upstream signals leading to TORC1 inhibition when wing discs reach their target size.

## Discussion

We study here how ecdysone regulates the size of *Drosophila* wing discs. We find that exogenous ecdysone supports wing disc proliferation in a concentration-dependent manner—higher levels of ecdysone support wing discs to grow to larger sizes. We find that the ability of ecdysone to promote disc cell proliferation depends on the size of the wing disc, with a given concentration of ecdysone promoting the proliferation of cells in a small wing disc but not in a large wing disc. These results are consistent with previous transplantation experiments whereby wing discs were implanted into the abdomens of adult female flies. This enabled the wing discs to proliferate to a certain size and then stop^4^. Since ecdysone levels in adult females are fairly stable, this might reflect the fact that every concentration of ecdysone can sustain wing proliferation only up to a certain maximal size. Important to point out is that in the Tub^ts^ > *spok*^*i*^ + 20E system that we use here the wing discs do not stop proliferating because of a limitation of space or nutrients, since *wts* and *TSC2* knockdown discs overproliferate massively (Figs. 3d, 4b–d). Also important is that the larval environment is competent to sustain proliferation because the wing discs do proliferate and grow robustly for more than 48 h before terminating.

We then tested which signaling pathways become limiting when a wing disc reaches the maximal size that can be supported by a given concentration of ecdysone. We do not observe a drop in Dpp or Yki signaling, and find that the wing pouch, which gives rise to the adult wing blade, terminates proliferation even in the absence of *brk* or *wts*. This indicates that neither Dpp signaling nor Hpo/Yki signaling determine the size at which the wing pouch stops proliferating for a given ecdysone concentration. Instead, termination of proliferation coincides with a drop in TORC1 activity, and is bypassed by reactivating TORC1. Indeed, wing pouch cells can proliferate in the absence of ecdysone signaling if TORC1 is activated genetically, indicating that TORC1 is epistatically downstream of ecdysone for wing cell proliferation. Cell growth and cell cycle progression are intertwined, so that cells usually stop growing if they stop proliferating, and the other way around. Interestingly, the finding that a drop in TORC1 activity controls the termination of proliferation implies that cell cycle progression in the pouch terminates because of a drop in cell growth, and not the other way around. These data fit with findings from the Struhl lab that reduced TORC1 signaling can prevent cell proliferating despite active Yki due to nuclear seclusion of Yki^45^. This drop in TORC1 activity occurs via an upstream signal which we have not yet identified, but will be the focus of future work.

What are the contributions of Dpp/Brk and Hpo/Yki signaling in tissue size determination outside the wing pouch? This is less clear. Both brk loss-of-function as well as yki hyperactivation can cause a bypass in proliferation termination outside the pouch. However, brk is present laterally both when cells in this region are proliferating and when proliferation stops. Previous reports have shown that lateral brk reduces the rate of proliferation in this region thereby equalizing proliferation throughout the wing disc^46,47^. Hence, we favor a model whereby Dpp/Brk signaling differentially modulates the rate of proliferation of different regions of the wing disc to give it a particular shape, but it does not determine when proliferation stops. Regarding yki, we do not see a drop in global yki activity in terminated discs compared to proliferating discs (Fig. 3c). A more detailed and spatially resolved analysis of yki activity in wing discs throughout larval third instar found that yki activity in proximal regions, if anything, increases as animals approach the end of larval development^36^, which would be inconsistent with yki turning off to terminate proliferation here. In sum, future work will be necessary to understand better whether Dpp/Brk or Hpo/Yki signaling regulates final tissue size downstream of ecdysone outside the pouch. The mechanisms regulating proliferation termination may be distinct in different regions of the disc.

Our data indicate that ecdysone levels set the target size of the wing disc (Fig. 2c), but the downstream effector mechanism that terminates cell proliferation is not caused by a change in ecdysone signaling, since we do not see ecdysone signaling either increasing or decreasing when wing discs stop proliferating (Fig. 2a). Although future studies will be required to understand this mechanistically, it means that ecdysone signaling levels and the physical size of the wing disc are getting integrated to determine whether the cells should continue proliferating or not. An alternate interpretation is that we have not found the optimal concentration of ecdysone feeding based on the target genes we assayed, thereby causing an artificial drop in TORC1 activity. Several observations speak against this interpretation. Firstly, wts mutant discs do continue proliferating, but not in the wing pouch, indicating there is no limitation to autonomous cell proliferation in our setup. Secondly, we performed a titration curve of ecdysone feeding, from very low levels that clearly are not able to support sufficient target gene expression, to the maximal ecdysone levels possible without inducing pupation, and obtained similar results at all these concentrations. Another limitation of our study is that EdU staining provides only a relative measurement of cell proliferation in a single time point.

While our manuscript was in revision, a paper was published by the Struhl lab that uses a similar setup as we do here, knocking down ecdysone synthesis in 3rd instar larvae^31^. Like us, they find that neither hyperactive Dpp signaling nor hyperactive yki signaling can maintain proliferation in the wing pouch. We discover here the limiting factor for proliferation in the wing pouch to be TORC1 and we show that ecdysone signaling affects this final size set-point in a concentration-dependent manner.

## Methods

### Fly strains and husbandry

*Spok* knockdown was achieved by crossing Tub-GAL4, Tub-GAL80^ts^/TM6B females to y,w; UAS-*spok* RNAi; UAS-*spok* RNAi males (kind gift from Mike O’ Connor). To collect experimental animals, flies were allowed to lay eggs in vials for roughly 8 h. Tubes were kept at 18 °C for 6 days to reach the L2/L3 molt. Non-TM6B, early L3 larvae were manually picked and transferred to vials containing food previously mixed with 20E (25 µM final concentration, unless otherwise indicated), and placed at 29 °C to induce *spok* RNAi expression. Every 48 h larvae were transferred into food supplemented with fresh 20E. AFG clones expressing *TSC2* RNAi were generated by heat-shocking animals at 32 °C for 45 min directly prior to 20E feeding and the 29 °C temperature shift. For rapamycin treatment larvae were fed food supplemented with 200 µM rapamycin for 4 h prior to disc dissection.

Gene knockdowns were achieved using the following KK lines: VDRC ID 101887 (*brk*), VDRC ID 106174 (*wts*), VDRC ID 103417 (*TSC2*), VDRC ID 103703 (*Akt*), VDRC ID 101538 (*GSK3ß*), VDRC ID 104369 (*S6K*) and Bloomington stocks 51789 (*brk*^*i*^ Trip), and 31770 (*TSC2*^*i*^ Trip). The following fly stocks were kindly provided by the following people: phmG4, Tub-GAL80ts from Kim Rewitz, UAS-arm^S10^ from Michael Boutros, UAS-CycD from Bruce Edgar, RagC S54N from Kun-Liang Guan and Ubx-FLP from Eugenia Piddini. UAS-Cdk4 was from FlyORF (stock 1652).

To knockdown *spok* using the lexA/lexAop system: Tubulin-LexA flies were a kind gift from Konrad Basler. To clone LexAop-*spok*^*i*^, 454nt of the *spok* coding region was PCR amplified from genomic DNA of UAS-*spok*-RNAi flies using OKH974 (ggTCTAGAcgtattttatgtgcta) / OKH975 (ccTCTAGAccgagctaaatttct), and this region was cloned twice in inverse orientation as XbaI fragments first into the AvrI site and second into the NheI site of the pWIZ vector, yielding pKH469. The LexAop-hsp70 promotor was PCR amplified from addgene plasmid #26224 using OKH981 (ggatgcatGCCGGGTCCTCAACGACA)/OKH982 (GCCAGTGCCAGTTCCTGAT) and subcloned as a NsiI/BglII fragment into the SbfI/BglII site of pKH469 yielding the LexAop-*spok*^*i*^ construct pKH472.

In order to express *TSC2*^*i*^ in the tub^*ts*^ > *spok*^*i*^ + 20E system (Supplementary Fig. 8a), *TSC2*^*i*^ (VDRC stock 103417, which contains two insertions, at 30B and 40D) was recombined with *spok*^*i*^. Progeny were screened by genomic PCR for the presence of the TSC2^i^ at 30B and *spok*^*i*^, and the absence of the *TSC2*^*i*^ 40D locus.

For genotyping stocks and recombinant chromosomes, oligos used are listed in Supplementary Table 1.

The genotypes of animals used in all figure panels are indicated in Supplementary Table 2.

### Quantifications

EdU, OPP, or pGSK3b signals were quantified using ImageJ by defining wing disc area via the presence of DAPI/nuclear stain, and then measuring the integrated density of the EdU, OPP or pGSK3b signal in this area. Quantification of lysotracker staining was done analogously, except the wing disc was outlined manually. The signal was then normalized to wing disc area. Note that this results in values with arbitrary units, and should not be used to compare signal levels between different experiments since the absolute value is influenced by staining and imaging settings. To measure pouch area, pouches were outlined using ImageJ and the area was quantified using the ‘measure’ tool. To measure nuclear vs cytosolic yki, an ImageJ macro was developed which allows the user to outline the pouch, and then the macro uses the DAPI channel to segment nuclei vs cytosol in quantifying the yki signal. The code is available at GitHub (https://github.com/aurelioteleman/Teleman-Lab/tree/master/ImageJ%20Macros).

### Generation of stronger UAS-spok RNAi lines

The original UAS-*spok*^*i*^ stock kindly provided by Michael O’Connor, contains two UAS-*spok*^*i*^ insertions, on II and III, both of which combined are required to efficiently knockdown *spok*. To generate single UAS-*spok* RNAi transgenes strong enough to efficiently knocks down *spok*, we first separated the two UAS-*spok* RNAi transgenes and transposed the insertion on chromosome II to a different locus using delta2-3 transposase. This yielded the stocks UAS-*spok* RNAi #4/Cyo and UAS-*spok* RNAi #8/TM6B. Supplementary Table 2 indicates which UAS-*spok* RNAi insertion is used in which figure panel.

### Immunoblotting and antibodies

Immunoblotting of discs lysates was performed as follows:^25^ To detect phosphorylation on S6K, wing discs were collected in batches of 10 discs per PCR tube containing Schneider’s medium on ice. Every batch was immediately spun down after disc collection and the disc pellet was kept on dry ice. Once sufficient wing disc batches were collected, they were successively lysed in one and the same lysis buffer by pipetting up and down and transferring the lysate to the next tube. Lysate was directly boiled at 95 °C for 5 min. Antibodies used are rabbit anti phospho-S6 kinase (T398) (PhosphoSolutions p1705-398) 1:1000, rabbit anti phospho-Akt (Ser505) (Cell signaling 4054) 1:1000, rabbit anti Akt 1:1000 (Cell signaling 9272), rabbit anti phospho-ERK1/2 1:1000 (Cell signaling 4370), rabbit anti ERK (Cell Signaling 9102) 1:2000, rabbit anti phospho-GSK3b (Ser9) (Cell signaling 9336) 1:1000, rabbit anti phospho-Smad1/5 (Ser463/465) (41D10) (Cell signaling 9516) 1:200, mouse anti rat CD2 (Linaris LFA-2) 1:200, rabbit anti phospho-ribosomal protein S6^42^ 1:1000, rabbit anti phospho-Akt Thr342^40^ 1:1000, rabbit anti phospho (S/T) Akt substrate (Cell signaling 9611) 1:1000, rabbit anti *Drosophila* cleaved caspase Dcp-1 (Cell signaling 9578) 1:200. Anti phospho-Yorkie (used 1:1000) and anti total-Yorkie (used 1:2000) antibodies were kind gifts from Nic Tapon^48^ and DJ Pan^49^. Mouse anti wg antibody was obtained from Hybridoma bank (1:200). Guinea pig anti brk (1:200) and guinea pig anti *Drosophila* S6K (1:2000) were generated by us and described and validated in the previous studies^42,50,51^.

### Quantitative RT-PCR

Oligos used for quantitative RT-PCR are listed in Supplementary Table 1. RNA was extracted using TRIZOL (Invitrogen) following manufacturer’s instructions and reverse-transcribed with Maxima H Minus Reverse Transcriptase (ThermoScientific) also following manufacturer’s instructions. Q-PCR was performed using Maxima SYBR Green/Rox (Fermentas), normalized to rp49, with an annealing temperature of 60 °C.

### Total protein quantification of wing discs

Per genotype three biological replicates were analyzed. Per biological replicate 6 wing discs were dissected into PCR tubes containing PBS. Discs were briefly spun down and PBS was removed leaving 10 µL behind. 10 µl of 12 M Urea (6 M final) was added and discs were lysed by pipetting up and down repeatedly. Disc lysates were split into two technical replicates and to 10 µL each 200 µl BCA reagent (Thermo Scientific) was added. BCA reaction mix was incubated for 30 min at 37 °C before measuring OD562.

### EdU and OPP incorporation reactions

Discs were incubated with EdU or OPP in M3 medium while rotating, and processed and quantified as follows:^25^ For combined EdU and antibody stainings, first EdU (25 µM final concentration) was incorporated into discs during 1 h rotation in M3 medium. Afterwards, discs were fixed in 4% paraformaldehyde/PBS for 20 min at RT. Discs were permeabilized in PBS/0.2% Triton (PBT) and blocked in PBT/0.1% BSA (BBT) for 30 min prior to binding of primary antibodies in BBT O/N at 4 °C. The next morning discs were washed in BBT for 1 h prior to secondary antibody incubation in BBT for 1 h at RT. After secondary antibody binding, discs were washed for 15 min in PBT before they were transferred to EdU click-IT reaction mix (100 µl for 12 discs) for 30 min at RT. Afterwards, discs were washed in PBT and nuclei were stained using DAPI before discs were equilibrated in glycerol mounting medium (160 ml glycerol, 20 ml 10× PBS, 0.8 g n-PG, 12 ml water).

### Lysotracker staining

Wing discs and fat bodies were incubated in Schneider’s medium containing 50 nM lysotracker red DND-99 (Invitrogen L7528) for 5 min with slight agitation protected from light. Discs were mounted in a large volume of Schneider’s medium on glass slides surrounded by other larval tissues such as mouth hooks or brains in order to prevent tissue damage.

### Statistics and data presentation

All bar graphs show mean values, with error bars representing standard deviation. Statistical significance was determined by two-tailed, unpaired, students *t*-test. Prior to the application of *t*-tests, normality of the data were tested using the Shapiro–Wilk test and Kolmogorov–Smirnov tests, except for Q-RT-PCR data where normality was assumed. Figures were prepared with Adobe Photoshop or Affinity Photo.

### Reporting summary

Further information on research design is available in the Nature Research Reporting Summary linked to this article.

## Supplementary information

Supplementary Information

Reporting Summary

## Data Availability

All data generated or analyzed during this study are included in this published article, in its supplementary information files, and in the Source Data files.

## Code availability

All custom software used in this study is available at GitHub: https://github.com/aurelioteleman/Teleman-Lab.
