# Supplementary material for: Ecdysone regulates Drosophila wing disc size via a TORC1 dependent mechanism
Source: Nat Commun. 2021 Nov 18;12:6684. doi: 10.1038/s41467-021-26780-0 (PMC8602387; doi:10.1038/s41467-021-26780-0)
Supplement: Supplementary file 1 — Supplementary Information [file 41467_2021_26780_MOESM1_ESM.pdf]

Supplementary Information

Ecdysone regulates Drosophila wing disc size via a TORC1 dependent mechanism

Strassburger et al.

Table of Contents

1.Cover Page .... p. 1

2. Supplementary Figures with Legends .... p. 2

4. Supplementary Table 1: Sequences of oligos used in this study .... p. 18

3. Supplementary Table 2: Genotypes of all figure panels .... p. 18

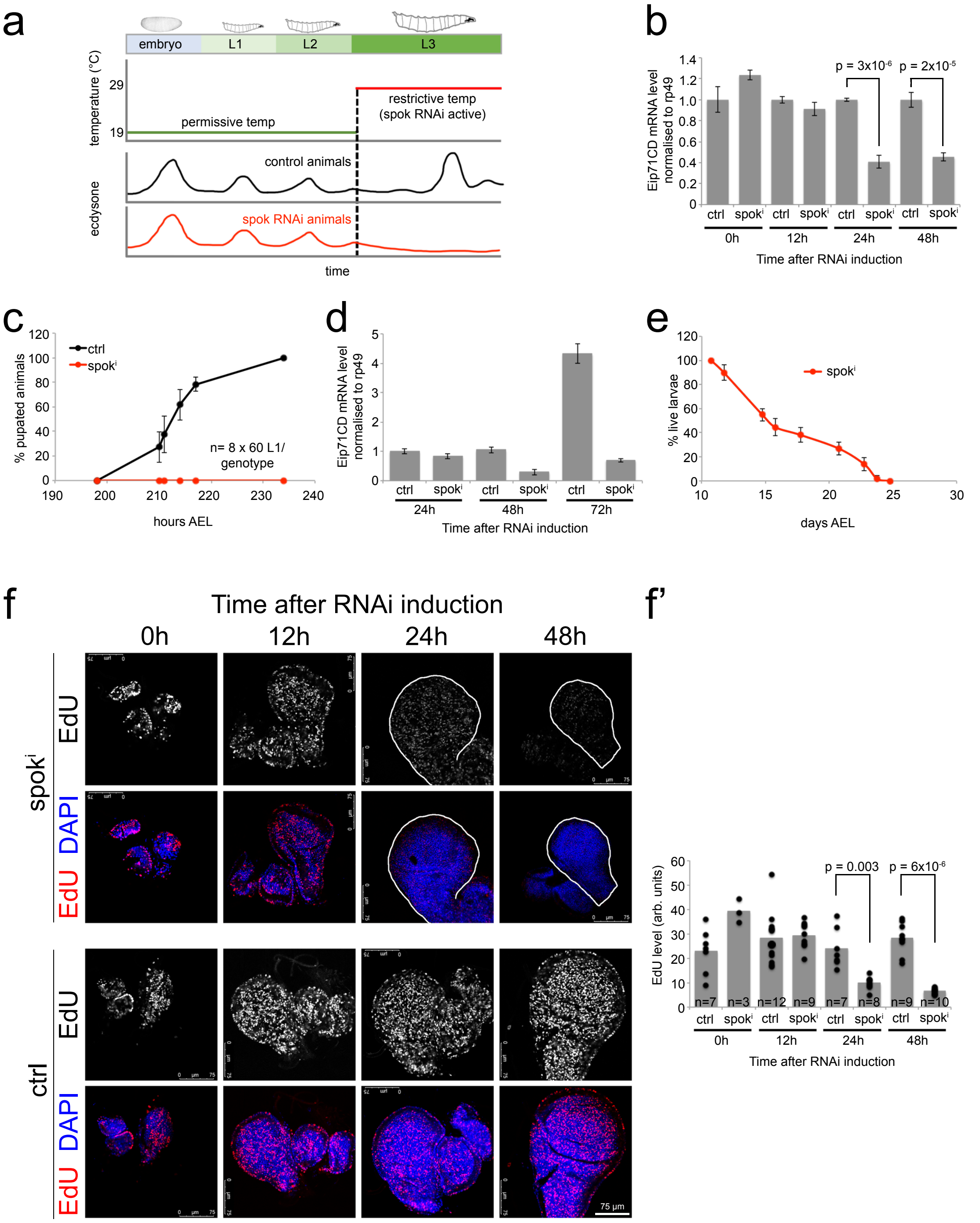

Supplementary Figure 1: Conditional knockdown of *spok* prolongs larval stage 3 by 2 weeks

(a) Schematic representation of the Tub^{ts}>*spok^{i}* system. Animals are kept at 19°C to allow *spok* expression and normal development until the 3rd instar (L3) stage. After 6 days, after the L2-L3 molt, animals are shifted to 29°C to induce *spok* knockdown, preventing the ecdysone pulse at the end of L3 that induces pupation.

(b) Knockdown of *spok* reduces ecdysone signaling. Ecdysone target gene *Eip71CD* mRNA levels, detected by Q-RT-PCR, normalized to *rp49*, from Tub^{ts}>+ (ctrl) or Tub^{ts} >*spok* RNAi (*spok^{i}*) larvae at 0-48 hours after knockdown induction. n=3 technical replicates x 2 biological replicates x 6 animals/sample. Bar = mean. Error bars = std. dev.

(c) Knockdown of *spok* prevents pupation. The percentage of pupated control (ctrl) or *spok^{i}* animals was determined over time. Error bars = std. dev.

(legend continues on next page)

(d) Knockdown of *spok* prevents the ecdysone pulse at the end of larval development. *Eip71CD* mRNA levels, detected by Q-RT-PCR, normalized to *rp49*, from control (ctrl) or $spok^i$ larvae at 24-72 hours after knockdown induction. n=3 technical replicates x 2 biological replicates x 6 animals/sample. Bar = mean. Error bars=std. dev.
(e) Tub^{ts}>$spok^i$ animals survive as active 3rd instar larvae for several weeks. The percentage of live larvae was counted over time. n=3 technical replicates x 2 biological replicates x 6 animals/sample. Error bars=std.dev.
(f-f') Knockdown of *spok* prevents wing disc proliferation. EdU incorporation of control (ctrl) or $spok^i$ wing discs assayed at 0-48h after knockdown induction. Representative images in (f), quantified in (f') Representative of 2 biological replicates.
For all panels p-values determined by t-test.

(figures continue on next page)

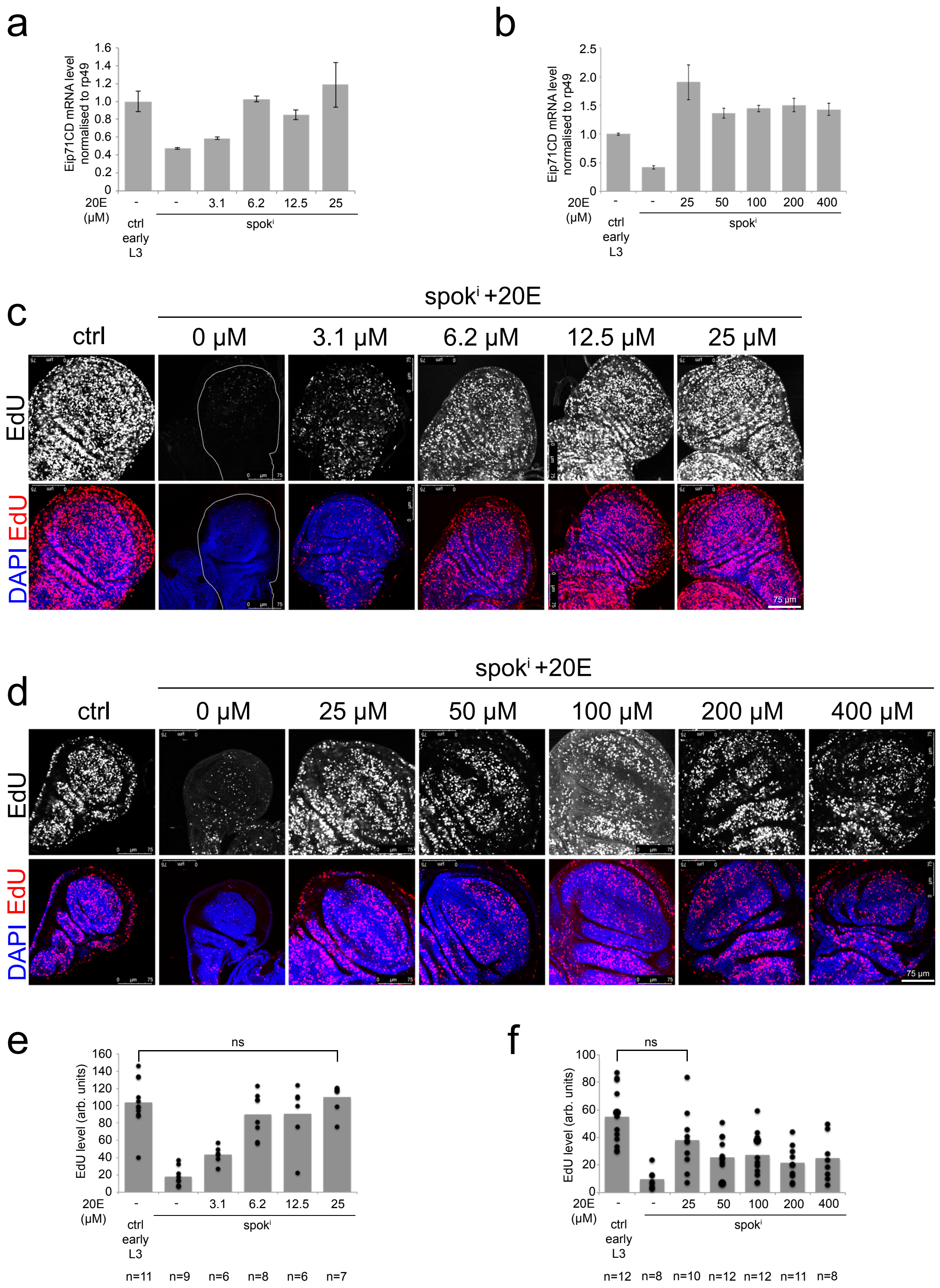

Supplementary Figure 2: Ecdysone feeding supports wing disc proliferation in *spok^i* animals

(a-b) 20E supplementation restores the expression of ecdysone target gene *Eip71CD*. *Eip71CD* mRNA levels detected by Q-RT-PCR, normalized to *rp49* for pre-wandering L3 ctrl larvae or larvae 48h after induction of *spok* knockdown with indicated concentrations of 20E supplemented into the food. (a-b) n=3 technical replicates x 2 biological replicates x 6 animals/sample. Bar = mean. Error bars = std. dev.

(c-f) 20E supplementation restores wing disc proliferation. EdU incorporation 48h after induction of *spok* knockdown and feeding of indicated concentrations of 20E. Representative images in c-d, quantified in e-f. Representative of 3 biological replicates. For all panels p-values determined by t-test.

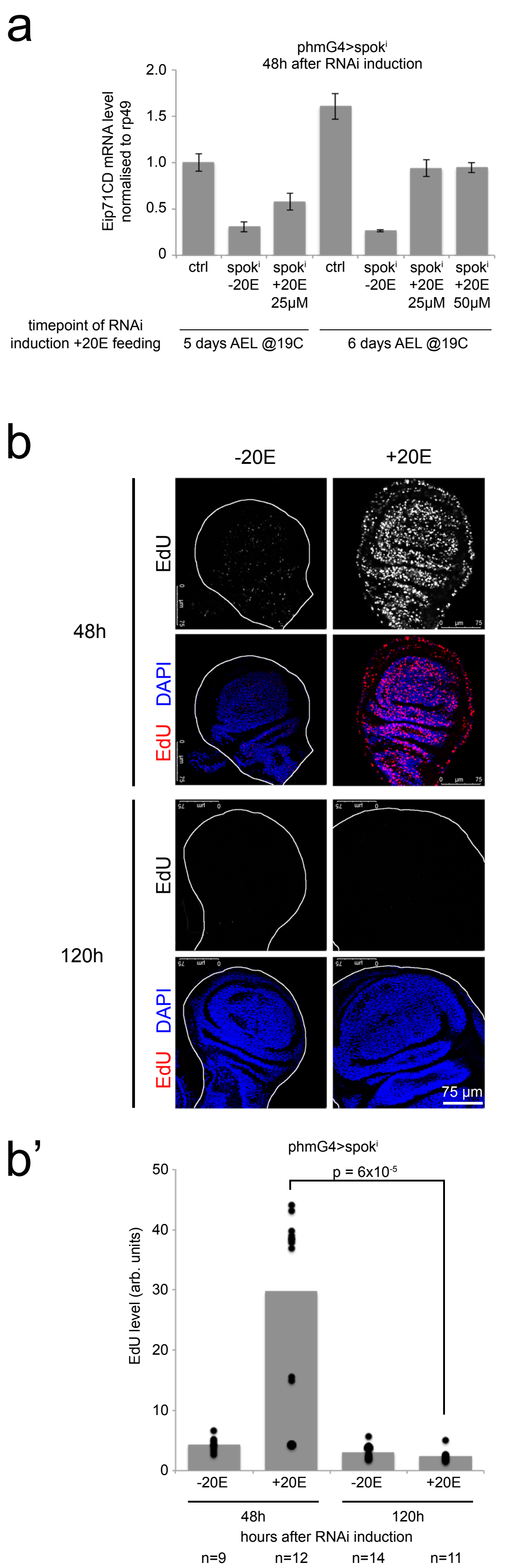

Supplementary Figure 3: Inducible knockdown of *spok* in the prothoracic gland recapitulates the phenotypes observed upon *spok* knockdown in the entire animal.

(legend continues on next page)

(a) Inducible knockdown of *spok* using phm-GAL4, Tub-GAL80^{ts}, UAS-*spok*-RNAi causes reduced ecdysone signaling in larvae 48h after RNAi induction, similar to the Tub^{ts}>*spok^{i}*

system. Animals were grown at 19C, and after 5 or 6 days, as indicated, *spok* RNAi was induced by shifting to 29C. At the same time, larval food was supplemented with the indicated concentrations of 20E. 48 hours after knockdown induction, ecdysone signaling was measured by detecting *Eip71CD* mRNA levels by Q-RT-PCR, normalized to *rp49*. ctrl: Tub>+ 48h after knockdown induction. n=3 technical replicates x 2 biological replicates x 6 animals/sample. Bar = mean. Error bars = std. dev.

(b-b') Similar to the Tub^{ts}>$spok^{i}$ +20E system, discs in animals with an inducible prothoracic gland-specific knockdown of *spok* (phm^{ts}>$spok^{i}$) that are fed 20E first proliferate for 48h and then terminate proliferation. By 120 hours after knockdown induction proliferation has stopped as judged by EdU staining. Representative images in (b), quantified in (b') Representative of 2 biological replicates. P-value determined by t-test.

(figures continue on next page)

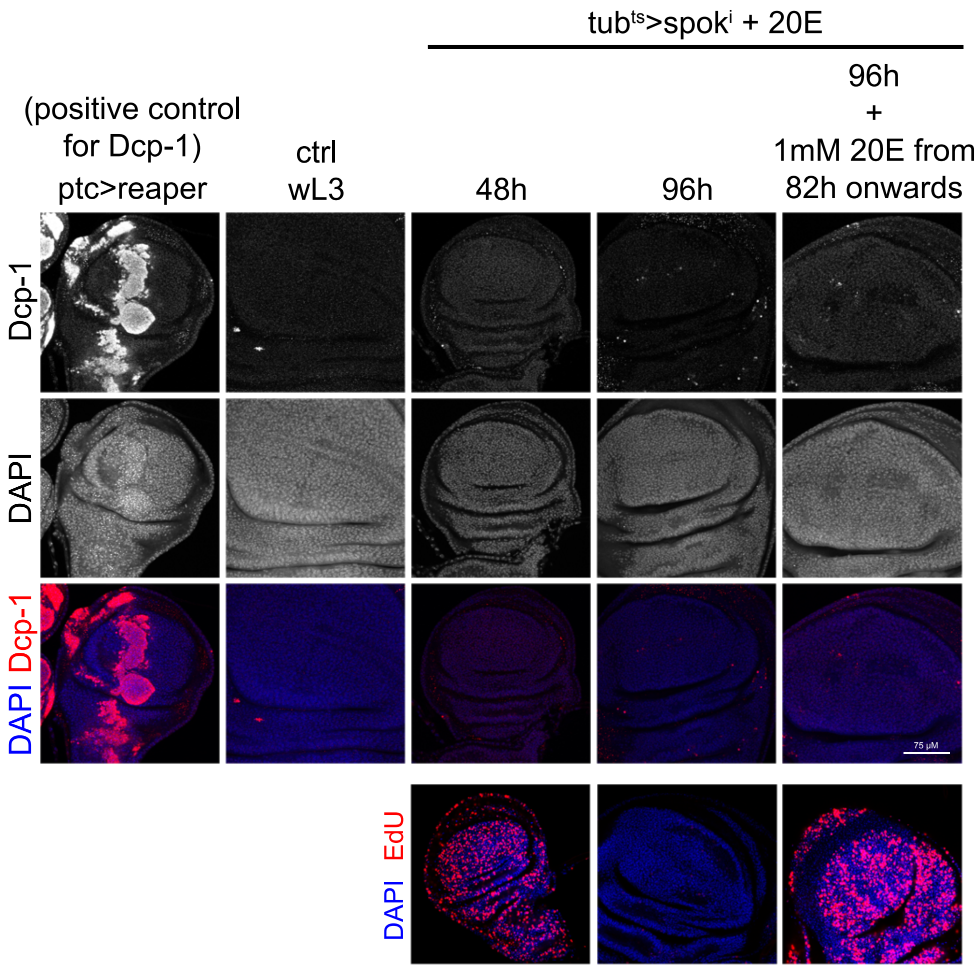

Supplementary Figure 4: Tub^{ts}>*spok^{i}* +20E discs are not apoptotic.
Cell death of Tub^{ts}>*spok^{i}* +20E discs was measured by immunostainings with dcp-1 antibody at different timepoints. As a positive control, reaper was expressed for 24h using ptcG4, Tub-GAL80^{ts}. Tub^{ts}>*spok^{i}* +20E discs have a few apoptotic cells, similar to w^{1118} wL3 wing discs which serve as a negative control. n = 8 discs/condition.

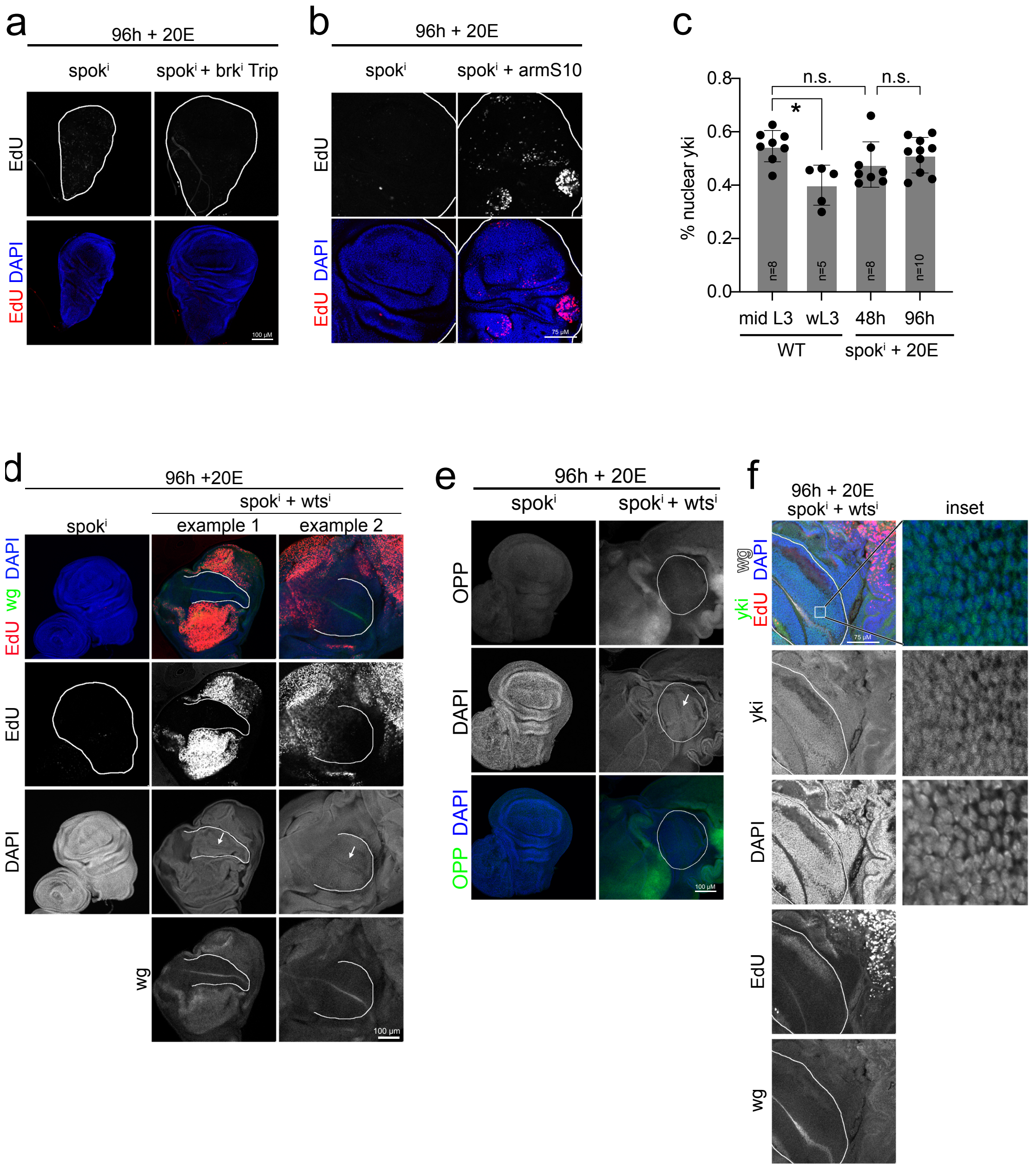

Supplementary Figure 5: *Dpp*, *hippo* and *wingless* signaling do not terminate proliferation of the wing pouch when the wing disc reaches the maximal target size supported by a given concentration of ecdysone.

(a) The wing pouch terminates proliferation despite knockdown of *brk* using a second RNA line (from the TRiP collection) independent of the one shown in main Figure 2b. Both Tub^{ts}>*spok*^{i} discs and Tub^{ts}>*spok*^{i}+*brk*^{i} discs have terminated proliferation by 96 hours after knockdown induction +20E, assayed by EdU incorporation. n = 16 discs.

(b) The wing pouch still terminates proliferation upon constitutive activation of wingless signaling by overexpression of armadillo^{S10}. The wing pouch of both Tub^{ts}>*spok*^{i} discs and Tub^{ts}>*spok*^{i}+*arm*^{S10} discs have terminated proliferation by 96 hours after knockdown induction +20E, assayed by EdU incorporation. n = 10 discs.

(c) Yki localization does not change when Tub^{ts}>*spok*^{i} +20E discs stop proliferating. WT and Tub^{ts}>*spok*^{i} discs were immunostained for yki and yki %nuclear localization was quantified in wing disc pouches. Statistical significance determined by t-test.

(legend continues on next page)

(d-e) Knockdown of *wts* in Tub^{ts}>*spok^{i}* +20E larvae (*spok^{i}* + *wts^{i}*) leads to overproliferation (d) and overgrowth (e) in proximal regions, assayed by EdU incorporation and OPP incorporation, respectively, whereas the pouch (white outline), identified by the ZNP double-stripe in DAPI staining (arrow) and a *wg* expression stripe, terminates proliferation. The degree of overproliferation in *spok^{i}* + *wts^{i}* discs varies: two representative examples are shown in (d) n = 6 discs.
(f) Knockdown of *wts* causes constitutive activation of yki. Yki localization is mainly nuclear in the wing pouch of *wts*-RNAi wing discs. The wing pouch (white outline) is identified morphologically and marked by the wg expression stripe. n = 10 discs.

(figures continue on next page)

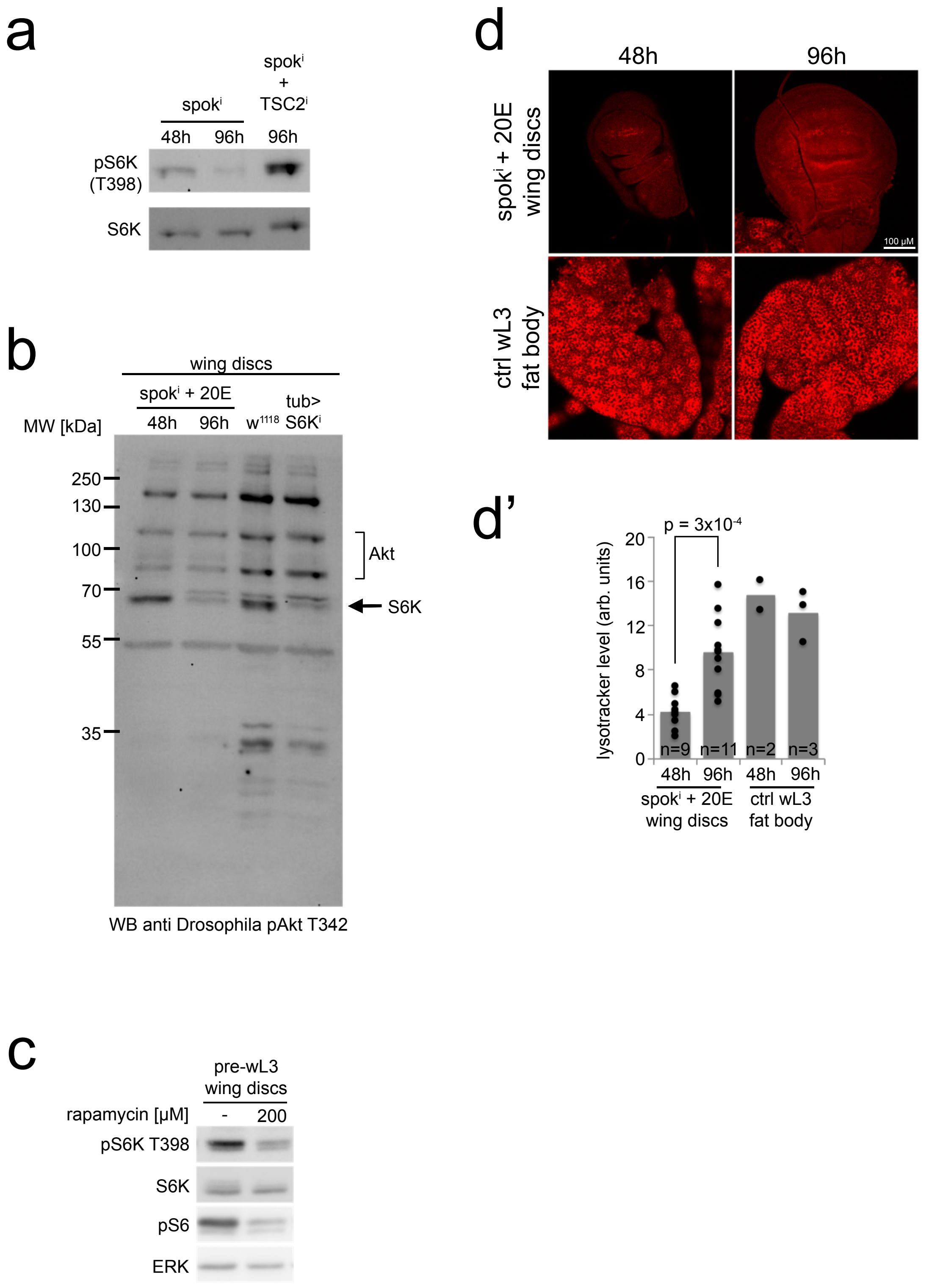

Supplementary Figure 6: TORC1 activity drops when Tub^{ts}>*spok^i* +20E discs terminate proliferation.

(a) TORC1 activity drops when Tub^{ts}>*spok^i* +20E discs terminate proliferation, and this is rescued by knockdown of TSC2. Phosphorylation of S6K (Thr398), a direct readout for TORC1 activity, was measured by immunoblotting lysates of wing discs from Tub^{ts}>*spok^i* +20E animals either 48h after knockdown induction, when they are still proliferating, or 96h after knockdown induction when they have terminated proliferation, or from Tub^{ts}>*spok^i* +TSC2^i +20E animals at 96h after knockdown induction. n=40 discs/condition x 4 biological replicates.

(legend continues on next page)

(b) Phosphorylation of S6K on Thr229 can be detected using an antibody that detects the very similar PDK1 sites on both Akt and S6K [54]. 96h after knockdown induction and 20E feeding Tub^{ts}>*spok*^{i} discs show a drop in pS6K Thr229. The S6K-specific band (arrow) was identified by comparing wing disc lysates of control (w^{1118}) and S6K RNAi (Tub>S6K^{i}) expressing larvae. **(c)** Feeding larvae rapamycin reduces S6K phosphorylation in wing discs, and shows that the lower band detected by the pS6K antibody does not respond to changes in TORC1 activity within physiological range. Pre-wandering L3 larvae were kept on food containing indicated amounts of rapamycin for 4h before wing disc lysates were subjected to immunoblotting with indicated antibodies. n = 20 discs/condition. (Note – same immunoblot as Suppl. Fig. 9f). **(d-d')** Consistent with TORC1 activity dropping, autophagy mildly increases in Tub^{ts}>*spok*^{i} +TSC2^{i} +20E discs 96h after knockdown induction and 20E feeding, measured by lysotracker staining. Representative pictures (d), quantification of lysotracker signal intensity (d'). Ctrl wL3 fat bodies were used as positive controls. P-value determined by t-test.

(figures continue on next page)

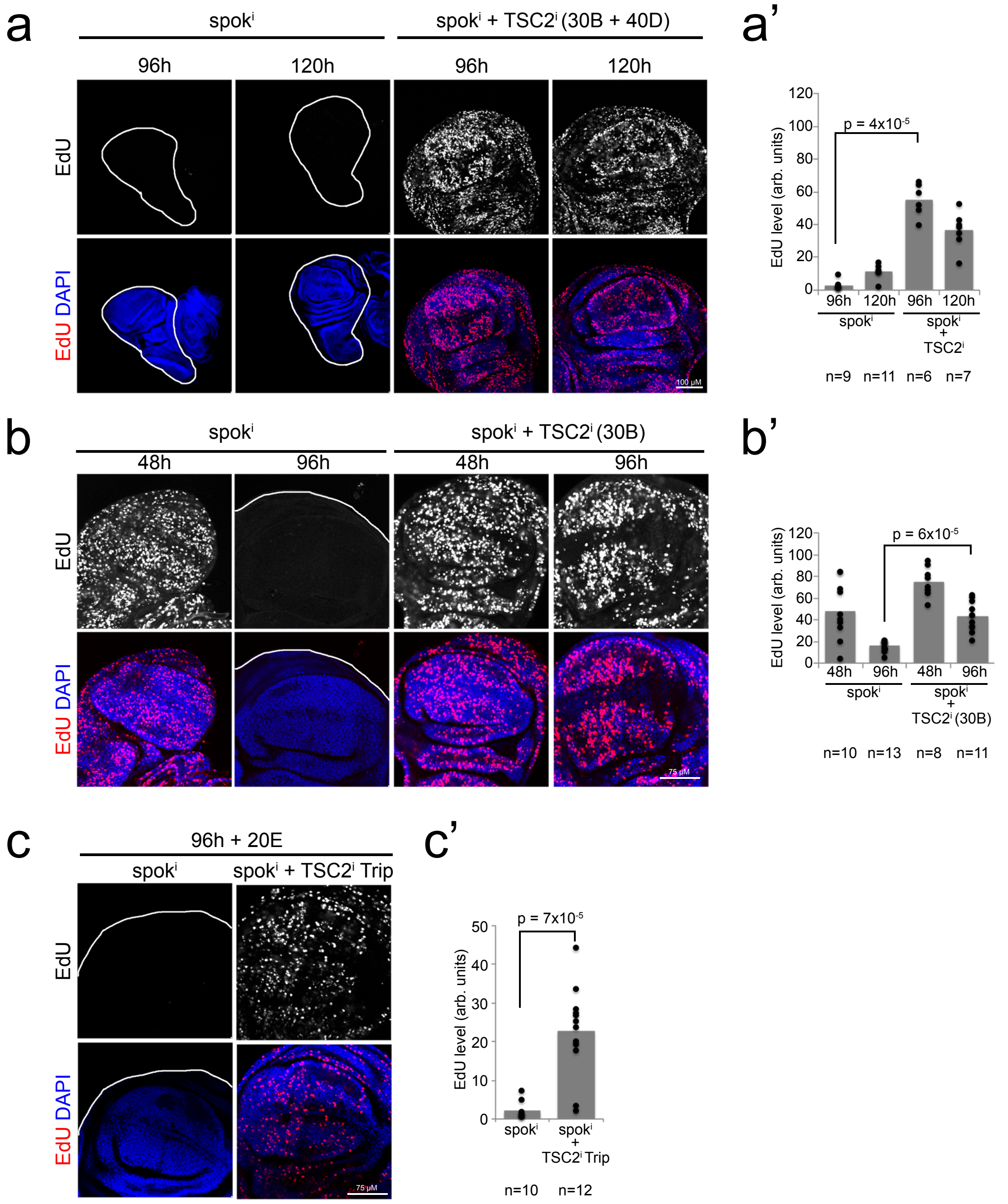

Supplementary Figure 7: Activation of TORC1 with different RNAi lines against TSC2 prevents proliferation termination in Tub^{ts}>*spok*^{i} + 20E discs.

(a-a') Tub^{ts}>*spok*^{i} +TSC2^{i} +20E discs stay EdU positive for at least 120h after knockdown induction and 20E feeding. The RNAi lines used here is the original line from the VDRC KK collection (number 103417) which contains two TSC2-RNAi insertions, at cytological locations 30B and 40D, which we verified by genomic PCR. Representative images (a), EdU quantification (a').

(b-b') Same as panel (a) except that the second insertion at the 40D locus was recombined away, assessed by genomic PCR. Knockdown with this line also rescues proliferation termination in Tub^{ts}>*spok*^{i} discs. Representative images (b), EdU quantification (b').

(c-c') An independent TSC2 RNAi line from the TRiP collection (Bloomington stock 31770) (Tub^{ts}>*spok*^{i} + *TSC2*^{i} Trip) also rescues proliferation termination. Representative images (c), EdU quantification (c'). For all quantifications p-values determined by t-test.

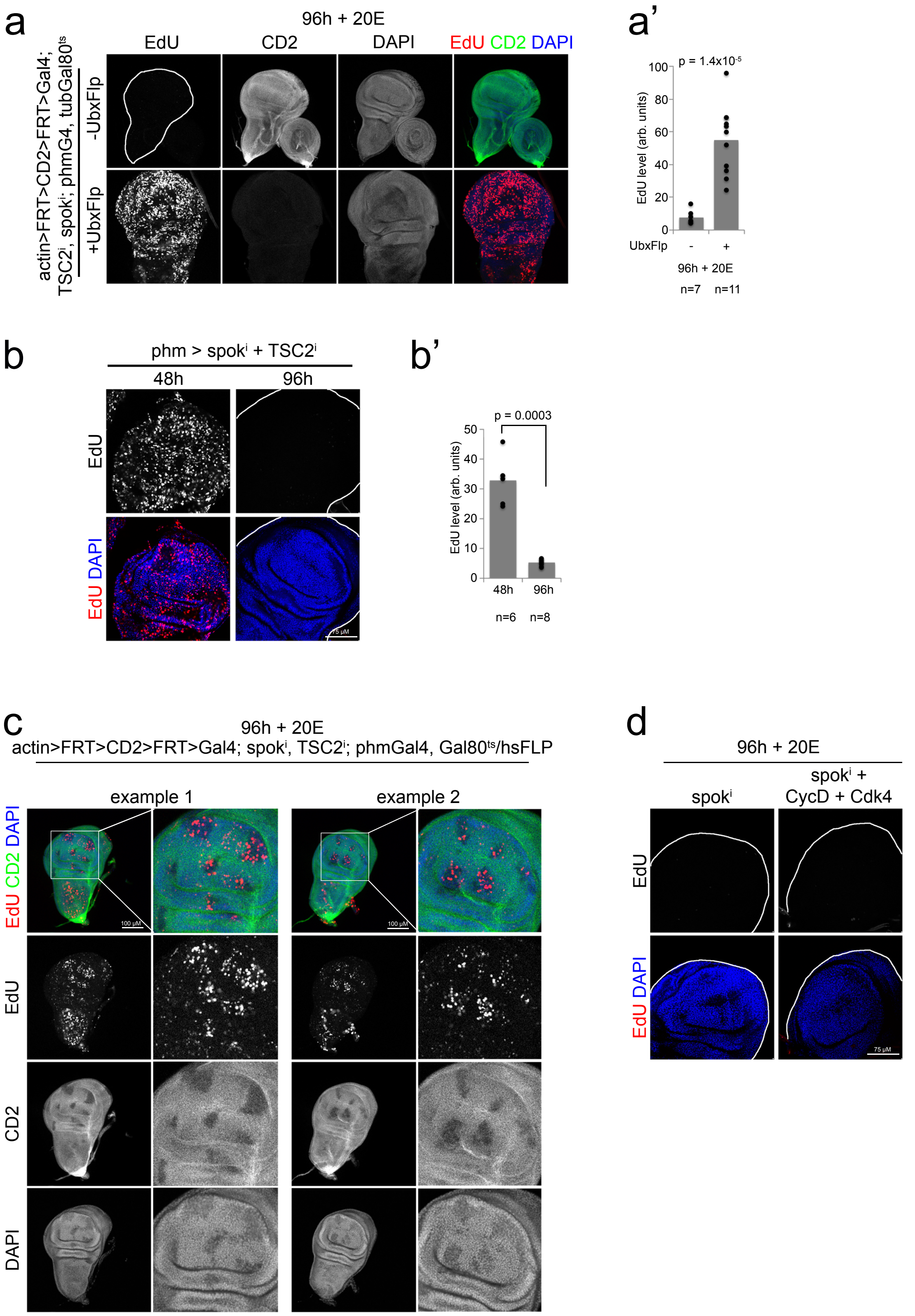

Supplementary Figure 8: Manipulation of TORC or Cdk4 activity in wing discs at proliferation termination.

(legend continues on next page)

(a-b') TSC2 knockdown in imaginal discs with UbxFLP bypasses the termination of proliferation of phm^{ts}>*spok^{i}* +20E discs. (a-a') Knockdown of TCS2 in both the prothoracic gland and imaginal discs using a combination of phmGAL4 and UbxFlp bypasses the termination of proliferation. Lack of staining anti CD2 shows efficient activation of actin-Gal4 driving TSC2^{i} expression in the presence of UbxFlp. Representative images (a), EdU quantification in (a'). Representative of 2 replicates. (b-b') As a negative control, knockdown of TSC2 in the prothoracic gland using phantom-GAL4 (phm^{ts}>*spok^{i}* + *TSC2^{i}*) does not prevent proliferation termination 96h after knockdown induction and 20E feeding. Representative images (b), EdU quantification (b').
(c) Clones in the wing disc expressing TSC2 RNAi bypass the termination of proliferation at 96h after spok knockdown and 20E feeding. *TSC2^{i}* expression was induced by heat shock (45 min, 32°C) and discs were stained for EdU and CD2 (marker for the FLP-mediated recombination event; CD2 negative cells express *TSC2^{i}*). n = 36 discs.
(d) Overexpression of Cyclin D + Cdk4 does not cause a bypass of proliferation termination 96h after spoki induction and 20E feeding. n = 9 discs. For all panels p-values determined by t-test.

(figures continue on next page)

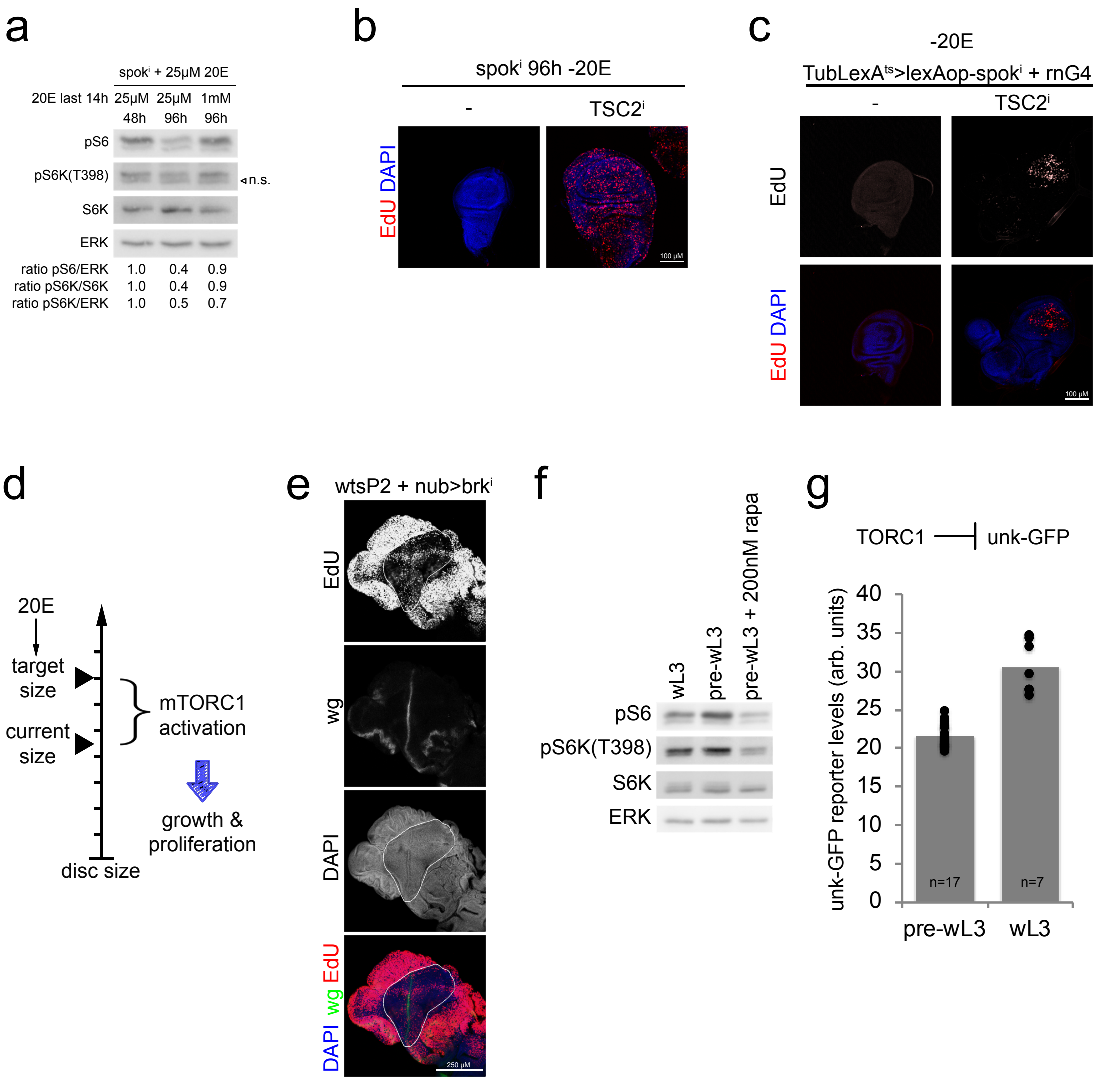

Supplementary Figure 9: TORC1 activity is downstream of ecdysone signaling

(a) TORC1 activity increases when the ecdysone concentration fed to animals is increased from 25μM to 1mM the last 14 hours prior to harvesting and lysing the wing discs for immunoblots. The band indicated as "n.s." in the pS6K panel does not decrease in wing discs treated with rapamycin (Suppl. Figure 6c).

(b-c) Knockdown of TSC2 enables wing disc cells to proliferate even in the absence of endogenous and exogenous ecdysone, indicating that TORC1 is one of the main factors downstream of ecdysone enabling cell proliferation. (b) Ubiquitous knockdown of TSC2 using Tub^{ts}>spok^{i} +/- TSC2^{i}, no 20E feeding, n = 10 discs. (c) Wing-pouch specific knockdown of TSC2 using the LexA system to knockdown spok and rnG4 to knockdown TSC2 in the pouch (LexA>lexAop spok^{i} +/- rnG4>UAS-TSC2^{i}), no 20E feeding. n = 10 discs.

(d) Schematic summary diagram illustrating the findings of this paper. A given concentration of ecdysone enables wing discs to grow only up to a certain maximal 'target' size. When wing discs reach that size, they stop proliferating and growing due to a drop in TORC1 signaling.

(e) The pouch (white outline, identified by wg staining) of wts^{P2} discs expressing brk^{i} under the control of nubbin-Gal4 terminate proliferation indicating that brk^{i} is dispensable for proliferation termination in the wing pouch also in the wts^{P2} system. n = 7 discs.

(f-g) TORC1 activity is reduced in wing discs of wandering L3 animals compared to pre-wandering animals, assayed (f) via immunoblotting wing disc lysates with pS6 and pS6K antibodies (rapamycin was used as a control in the 3rd lane) or (g) by quantifying levels of an unk-GFP reporter that is repressed by TORC1. (Note – panel f is the same immunoblot as Suppl. Fig. 6c).

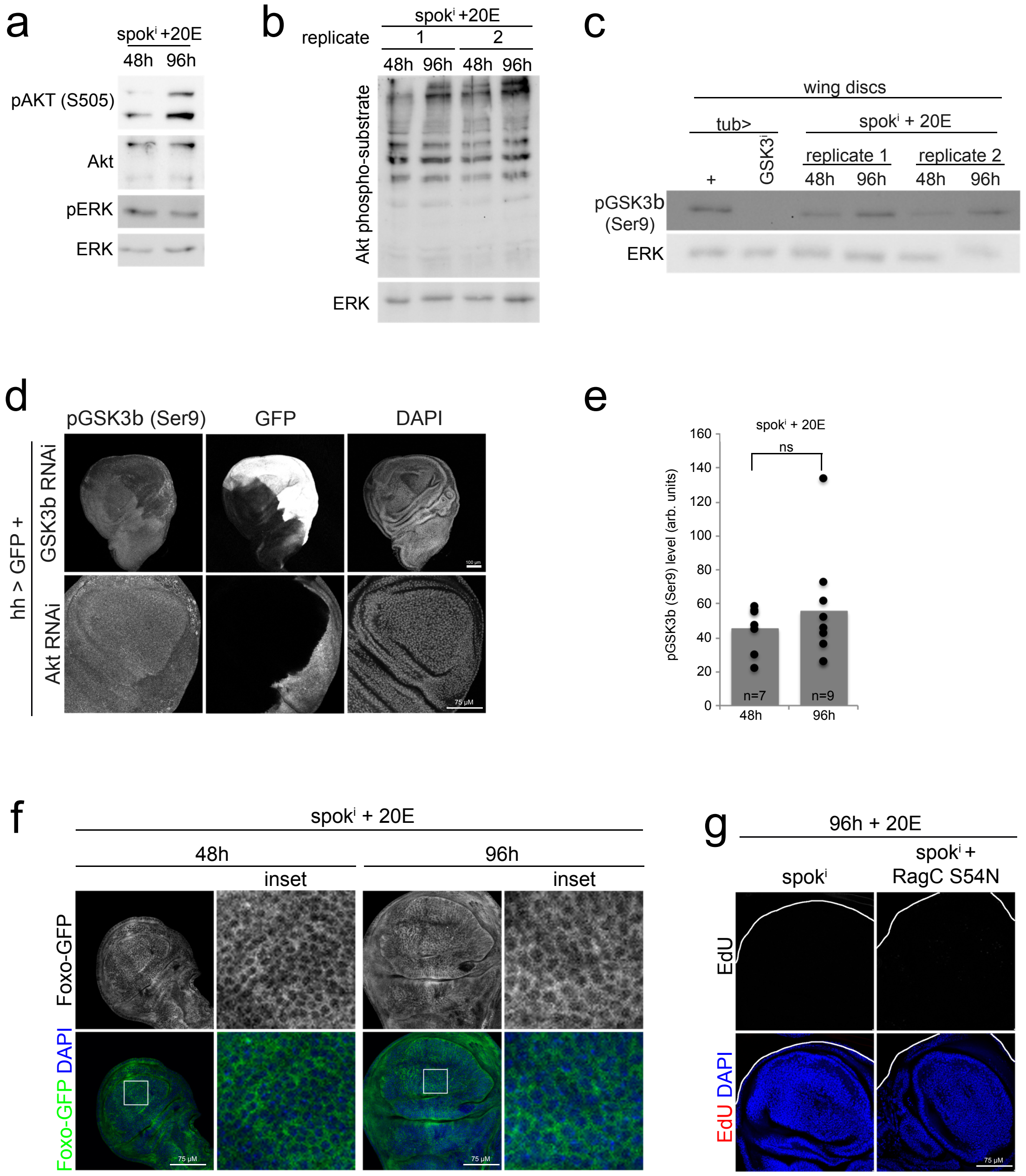

Supplementary Figure 10: Akt activity does not drop when Tub^{ts}>*spok^{i}* +20E discs terminate proliferation.

(a-c) Phosphorylation levels of ERK and Akt (TORC2 site Ser505) (a), of Akt substrates (b) and of GSK3ß (c) were determined by immunoblotting lysates of wing discs from Tub^{ts}>*spok^{i}* +20E (*spok^{i}*) animals either 48 or 96 hours after spok^{i} induction. In (c), knockdown of GSK3ß with Tub-G4 was used as a control to verify specificity of the detected band. n=3 biological replicates x 20 discs/sample.

(d) The phospho-GSK3ß antibody specifically detects Akt dependent GSK3ß phosphorylation by immunostaining in wing discs, since pGSK3ß staining levels are reduced when either GSK3ß (top panel) or Akt were knocked-down in the posterior compartment using hedgehog(hh)-Gal4. GFP indicates hh expression domain. n = 5 discs.

(e) Phosphorylation levels of GSK3ß were determined by immunostaining wing discs from Tub^{ts}>*spok^{i}* +20E animals either 48 or 96 hours after knockdown induction and quantifying integrated density normalized to wing disc area. Statistical significance determined by t-test.

(f) Foxo localization does not change when Tub^{ts}>*spok^{i}* +20E discs terminate proliferation. Foxo-GFP remains cytoplasmic at 48 and 96 hours after knockdown induction. n = 4 discs.

(g) Overexpression of constitutively active RagC does not bypass proliferation termination. Wing discs from Tub^{ts}>*spok^{i}* +RagC^{S54N} +20E larvae stop proliferating 96h after knockdown induction and 20E feeding. n = 13 discs.

Supplementary Table 1: Sequences of oligos used in this study.

For genotyping		
	forward	reverse
spoki	CAAGCGCAGCTGAACAAG	GGCACACTCGCTGCATAGT
40D	GCCCACTGTCAGCTCTCAAC	TGTAAAACGACGGCCAGT
30B	GCTGGCGAACTGTCAATCAC	TGTAAAACGACGGCCAGT
wtsP2	CTGCCGCCTGTTTTGAC	AGCGGCTGATGTTGAACTG
Gal4	GATTGACTCGGCAGCTCATC	TGGAACCTGACTCGAAGACC
Gal80	TGGGCAATCAAGACACATT	GCAAGGGCCCATTCTACGA
CycD	CAAGCGCAGCTGAACAAG	TCGCCGGTGAGGACATTTG
CDK4	CAAGCGCAGCTGAACAAG	CCGTCGCGCTCCAGAAAC
For quantitative RT-PCR		
	forward	reverse
Eip71CD	GCGTACCACCAGAAGTATAGGT	AGATTCGGCCATGTCAGCAG
Eip74B	ATCGGCGGCCTACAAGAAG	TCGATTGCTTGACAATAGGAATTTC
Ftz-f1	TGCGAGTCCTGCAAGGGATTCTTCA	GCTCGAACAGCCTCTAGCTTCATGC
rp49	GCTAAGCTGTCGCACAAA	TCCGGTGGGCAGCATGTG

Supplementary Table 2: Genotypes for all figure panels.

Figure	Label	Genotype
1	ctrl	w; +/+; tubG4, tubG80ts/+
	spoki	w; UAS-spok RNAi/+; tubG4, tubG80ts/UAS-spok RNAi
2	ctrl	w; +/+; tubG4, tubG80ts/+
	spoki	w; UAS-spok RNAi/+; tubG4, tubG80ts/UAS-spok RNAi
3	spoki	w; UAS-spok RNAi/+; tubG4, tubG80ts/UAS-spok RNAi
3b	spoki + brki	w; UAS-spok RNAi, UAS-Flp/dpp-FRT-CA, UAS-brk RNAi; tubG4, tubG80ts/UAS-spok RNAi
3c	spoki + wtsi	w; UAS-spok RNAi, UAS-wts RNAi/+; tubG4, tubG80ts/UAS-spok RNAi
3d-e	spoki + wtsP2	w; UAS-spok RNAi/+; tubG4, tubG80ts, wtsP2/wtsP2, UAS-spok RNAi
3f	wtsP2	w; +/+; wtsP2/wtsP2 (III)
4	ctrl	w; +/+; tubG4, tubG80ts/+
	spoki	w; UAS-spok RNAi/+; tubG4, tubG80ts/UAS-spok RNAi
4b-d	spoki + TSC2i	w; UAS-spok RNAi, UAS-TSC2 RNAi/+; tubG4, tubG80ts/UAS-spok RNAi
4e	TubtsLexA>lexAop spoki -	tubLexA/+; lexAop-spok RNAi/rnG4, tubG80ts (III)
	TubtsLexA>lexAop spoki rn>TSC2i	tubLexA/+; UAS-TSC2 RNAi/+; lexAop-spok RNAi/rnG4,tubG80ts
4f	wtsP2	w; +/+; wtsP2/wtsP2 (III)

4g	wtsP2	w; +/+; wtsP2/wtsP2 (III)
	wtsP2, nub>TSC2i	w; nubG4/UAS-TSC2 RNAi; wtsP2/wtsP2
Suppl. 1	ctrl	w; +/+; tubG4, tubG80ts/+
	spoki	w; UAS-spok RNAi/+; tubG4, tubG80ts/UAS-spok RNAi
Suppl. 2	ctrl	w; +/+; tubG4, tubG80ts/+
	spoki	w; UAS-spok RNAi/+; tubG4, tubG80ts/UAS-spok RNAi
Suppl. 3	ctrl	w; +/+; phmG4, tubG80ts/+
	spoki	w; UAS-spok RNAi/+; phmG4, tubG80ts/UAS-spok RNAi
Suppl. 4	ptc>reaper	w; ptcG4, tubG80ts/+; UAS-reaper/+ ; kept at 29°C 24h prior to dissection
Suppl. 5a	spoki	w; UAS-spok RNAi #4/+; tubG4, tubG80ts/+
	spoki + brki Trip	w; UAS-spok RNAi #4/+; tubG4, tubG80ts/UAS-brk RNAi Trip
Suppl. 5b	spoki	w; UAS-spok RNAi/+; tubG4, tubG80ts/UAS-spok RNAi
	spoki + armS10	w; UAS-spok RNAi/UAS-armS10; tubG4, tubG80ts/UAS-spok RNAi
Suppl. 5c	WT	w1118
	spoki	w; UAS-spok RNAi/+; tubG4, tubG80ts/UAS-spok RNAi
Suppl. 5d-f	spoki	w; UAS-spok RNAi/+; tubG4, tubG80ts/UAS-spok RNAi
	spoki + wtsi	w; UAS-spok RNAi, UAS-wts RNAi/+; tubG4, tubG80ts/UAS-spok RNAi
Suppl. 6	spoki	w; UAS-spok RNAi/+; tubG4, tubG80ts/UAS-spok RNAi
Suppl. 6a	spoki + TSC2i	w; UAS-spok RNAi, UAS-TSC2 RNAi/+; tubG4, tubG80ts/UAS-spok RNAi
Suppl. 6b	tub>S6Ki	w; UAS-S6K RNAi/+; tubG4, tubG80ts/+ kept at 29°C
Suppl. 6c-d	ctrl wL3	w1118
Suppl. 7a	spoki	w; UAS-spok RNAi/+; tubG4, tubG80ts/UAS-spok RNAi
	spoki + TSC2i (30B + 40D)	w; UAS-spok RNAi, UAS-TSC2 RNAi/+; tubG4, tubG80ts/UAS-spok RNAi
Suppl. 7b	spoki	w; UAS-spok RNAi #4/+; tubG4, tubG80ts/+
	spoki + TSC2i (30B)	w; UAS-spok RNAi #4, UAS-TSC2 RNAi (30B)/+; tubG4, tubG80ts/+
Suppl. 7c	spoki	w; UAS-spok RNAi #4/+; tubG4, tubG80ts/+
	spoki + TSC2i Trip	w; UAS-spok RNAi #4/+; tubG4, tubG80ts/TSC2 RNAi Trip
Suppl. 8a	-Ubx	actin>FRT>CD2>FRT>Gal4/y; UAS-TSC2 RNAi, UAS-spok RNAi #4/+; phmG4, tubG80ts/+
	+Ubx	actin>FRT>CD2>FRT>Gal4/UbxFlp; UAS-TSC2 RNAi, UAS-spok RNAi #4/+; phmG4, tubG80ts/+
Suppl. 8b	phm>spoki + TSC2i	w; UAS-spok RNAi, UAS-TSC2 RNAi/+; phmG4, tubG80ts/UAS-spok RNAi
Suppl. 8d	spoki	w; UAS-spok RNAi/+; tubG4, tubG80ts/UAS-spok RNAi
	spoki + CycD + Cdk4	w; UAS-spok RNAi, UAS-CycD; UAS-spok RNAi, UAS-Cdk4/tubG4, tubG80ts

Suppl. 9a	spoki	w; UAS-spok RNAi/+; tubG4, tubG80ts/UAS-spok RNAi
Suppl. 9b	spoki + TSC2i	w; UAS-spok RNAi, UAS-TSC2 RNAi/+; tubG4, tubG80ts/UAS-spok RNAi
Suppl. 9c	TubLexAts>LexAop-spoki + rnG4 +/- TSC2i	tubLexA/+; UAS-TSC2 RNAi/+; lexAop-spok RNAi/rnG4,tubG80ts
Suppl. 9e	wtsP2 + nub>brki	w; nubG4/UAS-brk RNAi; wtsP2/wtsP2
Suppl. 9f	wL3	w1118
Suppl. 9g	unk-GFP	unk-GFP
Suppl. 10a-c,e	spoki	w; UAS-spok RNAi/+; tubG4, tubG80ts/UAS-spok RNAi
Suppl. 10c	tub>GSK3i	w; UAS-S6K RNAi/+; tubG4, tubG80ts/+ kept at 29°C
Suppl. 10d	hh>GFP, GSK3ß RNAi	w; UAS-GFP/UAS-GSK3ß RNAi; hhG4/+
	hh>GFP, Akt RNAi	w; UAS-GFP/UAS-Akt RNAi; hhG4/+
Suppl. 10f	spoki + Foxo-GFP	w; UAS-spok RNAi/+; UAS-spok RNAi, UAS-Foxo-GFP/tubG4, tubG80ts
Suppl. 10g	spoki + RagC S54N	w; UAS-spok RNAi/+; UAS-spok RNAi, UAS-RagC S54N/tubG4, tubG80ts
