# Supplementary material for: Ecdysone regulates Drosophila wing disc size via a TORC1 dependent mechanism
Source: Nat Commun. 2021 Nov 18;12:6684. doi: 10.1038/s41467-021-26780-0 (PMC8602387; doi:10.1038/s41467-021-26780-0)
Supplement: Supplementary file 2 — Reporting Summary [file 41467_2021_26780_MOESM2_ESM.pdf]

nature research

Corresponding author(s): Aurelio Teleman

Last updated by author(s): 22/07/2021

Reporting Summary

Nature Research wishes to improve the reproducibility of the work that we publish. This form provides structure for consistency and transparency in reporting. For further information on Nature Research policies, see Authors & Referees and the Editorial Policy Checklist .

Statistics

For all statistical analyses, confirm that the following items are present in the figure legend, table legend, main text, or Methods section.

n/a	Confirmed	
☐	☒	The exact sample size (*n*) for each experimental group/condition, given as a discrete number and unit of measurement
☐	☒	A statement on whether measurements were taken from distinct samples or whether the same sample was measured repeatedly
☐	☒	The statistical test(s) used AND whether they are one- or two-sided<br>*Only common tests should be described solely by name; describe more complex techniques in the Methods section.*
☒	☐	A description of all covariates tested
☐	☒	A description of any assumptions or corrections, such as tests of normality and adjustment for multiple comparisons
☐	☒	A full description of the statistical parameters including central tendency (e.g. means) or other basic estimates (e.g. regression coefficient) AND variation (e.g. standard deviation) or associated estimates of uncertainty (e.g. confidence intervals)
☐	☒	For null hypothesis testing, the test statistic (e.g. *F*, *t*, *r*) with confidence intervals, effect sizes, degrees of freedom and *P* value noted<br>*Give P values as exact values whenever suitable.*
☒	☐	For Bayesian analysis, information on the choice of priors and Markov chain Monte Carlo settings
☒	☐	For hierarchical and complex designs, identification of the appropriate level for tests and full reporting of outcomes
☒	☐	Estimates of effect sizes (e.g. Cohen's *d*, Pearson's *r*), indicating how they were calculated

Our web collection on statistics for biologists contains articles on many of the points above.

Software and code

Policy information about availability of computer code

Data collection	The software running the Leica confocal microscope was Leica Application Suite X (LAS X) version 2.0.1.14392, which is commercially available.
Data analysis	All software used in this study is publicly or commercially available: Images were analyzed with ImageJ (1.47v) using scripts that have been uploaded to GitHub (https://github.com/aurelioteleman/Teleman-Lab). Figure preparation: Adobe Photoshop CS6 version (13.0 x64) or Affinity photo (1.10.1). Data analysis with Microsoft Excel (v16.16.6) or Graphpad Prism (9.0.2).

For manuscripts utilizing custom algorithms or software that are central to the research but not yet described in published literature, software must be made available to editors/reviewers. We strongly encourage code deposition in a community repository (e.g. GitHub). See the Nature Research guidelines for submitting code & software for further information.

Data

Policy information about availability of data

All manuscripts must include a data availability statement. This statement should provide the following information, where applicable:

- Accession codes, unique identifiers, or web links for publicly available datasets
- A list of figures that have associated raw data
- A description of any restrictions on data availability

All data generated or analysed during this study are included in this published article, in its supplementary information files, and in the Source Data files.

Field-specific reporting

Please select the one below that is the best fit for your research. If you are not sure, read the appropriate sections before making your selection.

Life sciences study design

All studies must disclose on these points even when the disclosure is negative.

Sample size	No sample size calculations were performed. Instead, all experiments were performed on at least 6 animals per condition, and the entire experiment was repeated at least 2 times, which from experience is sufficient to assure that the selected examples are representative.
Data exclusions	No data were excluded.
Replication	Except where noted in the figure legends, experiments were replicated at least 2 times. All attempts at replication were successfull for the data that are included in this manuscript.
Randomization	All animals were randomly alloted.
Blinding	The experiments were not blinded because it is not standard in the field.

Reporting for specific materials, systems and methods

We require information from authors about some types of materials, experimental systems and methods used in many studies. Here, indicate whether each material, system or method listed is relevant to your study. If you are not sure if a list item applies to your research, read the appropriate section before selecting a response.

Materials & experimental systems

n/a	Involved in the study	
☐	☒	Antibodies
☒	☐	Eukaryotic cell lines
☒	☐	Palaeontology
☐	☒	Animals and other organisms
☒	☐	Human research participants
☒	☐	Clinical data

Methods

n/a	Involved in the study	
☒	☐	ChIP-seq
☒	☐	Flow cytometry
☒	☐	MRI-based neuroimaging

Antibodies

Antibodies used	As described in the Materials & Methods section: Antibodies used are rabbit anti phospho-S6 kinase (T398) (PhosphoSolutions p1705-398) 1:1000, rabbit anti phospho-Akt (Ser505) (Cell signaling 4054) 1:1000, rabbit anti Akt 1:1000 (Cell signaling 9272), rabbit anti phospho-ERK1/2 1:1000 (Cell signaling 4370), rabbit anti ERK (Cell Signaling 9102) 1:2000, rabbit anti phospho-GSK3b (Ser9) (Cell signaling 5558) and rabbit anti phospho-Smad1/5 (Ser463/465) (41D10) (Cell signaling 9516) 1:200, mouse anti rat CD2 (Linaris LFA-2) 1:200, rabbit anti phospho-ribosomal protein S6 1:1000, rabbit anti phospho-Akt Thr342 1:1000, rabbit anti phospho (S/T) Akt substrate (Cell signaling 9611) 1:1000, rabbit anti Drosophila cleaved caspase Dcp-1 (Cell signaling 9578) 1:200, Anti phospho-Yorkie (used 1:1000) and anti total-Yorkie (used 1:2000) antibodies were from Nic Tapon and DJ Pan. Mouse anti wg antibody was obtained from Hybridoma bank (1:200). Guinea pig anti brk (1:200) and guinea pig anti Drosophila S6K (1:2000) were generated by us.
Validation	Our home-made antibodies were validated in the following publications:<br>phospho-S6k: PMID 26988032<br>brk: PMID 23337628<br>total S6K: PMID 20444422<br>phospho-S6 PMID 32589966<br>The commercial antibodies are all widely used in the field and are validated on the corresponding webpages:<br>phospho-Akt: by WB on manufacturer's website +- wortmannin<br>total Akt: by WB on manufacturer's website +- siRNA knockdown<br>phospho-ERK: by WB on manufacturer's website +- U0126<br>total ERK: by WB on manufacturer's website +-siRNA knockdown<br>phospho-GSK3b: by WB on manufacturer's website +- PDGF<br>phospho-Smad1/5: by WB on manufacturer's website +- BMP4<br>CD2: tissue immunostaining by us on wing discs +- CD2 expression construct<br>phospho-Yorkie: PMID 20159599<br>total-Yorkie: PMID 16096061

Animals and other organisms

Policy information about studies involving animals; ARRIVE guidelines recommended for reporting animal research

Laboratory animals

Species: Drosophila melanogaster
Sex: mixed males and females.
Age: specified in the figures
Strains: Full genotype for each figure panel is provided in Suppl. Table 1:
1 ctrl w; +/+; tubG4, tubG80ts/+
spoki w; UAS-spok RNAi/+; tubG4, tubG80ts/UAS-spok RNAi
2 ctrl w; +/+; tubG4, tubG80ts/+
spoki w; UAS-spok RNAi/+; tubG4, tubG80ts/UAS-spok RNAi
3 spoki w; UAS-spok RNAi/+; tubG4, tubG80ts/UAS-spok RNAi
3b spoki + brki w; UAS-spok RNAi, UAS-Flp/dpp-FRT-CA, UAS-brk RNAi; tubG4, tubG80ts/UAS-spok RNAi
3c spoki + wtsi w; UAS-spok RNAi, UAS-wts RNAi/+; tubG4, tubG80ts/UAS-spok RNAi
3d-e spoki + wtsP2 w; UAS-spok RNAi/+; tubG4, tubG80ts, wtsP2/wtsP2, UAS-spok RNAi
3f wtsP2 w; +/+; wtsP2/wtsP2 (III)
4 ctrl w; +/+; tubG4, tubG80ts/+
spoki w; UAS-spok RNAi/+; tubG4, tubG80ts/UAS-spok RNAi
4b-d spoki + TSC2i w; UAS-spok RNAi, UAS-TSC2 RNAi/+; tubG4, tubG80ts/UAS-spok RNAi
4e TubtsLexA>lexAop spoki - tubLexA/+; lexAop-spok RNAi/rnG4, tubG80ts (III)
TubtsLexA>lexAop spoki rn>TSC2i tubLexA/+; UAS-TSC2 RNAi/+; lexAop-spok RNAi/rnG4,tubG80ts
4f wtsP2 w; +/+; wtsP2/wtsP2 (III)
4g wtsP2 w; +/+; wtsP2/wtsP2 (III)
wtsP2, nub>TSC2i w; nubG4/UAS-TSC2 RNAi; wtsP2/wtsP2
Suppl. 1 ctrl w; +/+; tubG4, tubG80ts/+
spoki w; UAS-spok RNAi/+; tubG4, tubG80ts/UAS-spok RNAi
Suppl. 2 ctrl w; +/+; tubG4, tubG80ts/+
spoki w; UAS-spok RNAi/+; tubG4, tubG80ts/UAS-spok RNAi
Suppl. 3 ctrl w; +/+; phmG4, tubG80ts/+
spoki w; UAS-spok RNAi/+; phmG4, tubG80ts/UAS-spok RNAi
Suppl. 4 ptc>reaper w; ptcG4, tubG80ts/+; UAS-reaper/+ ; kept at 29°C 24h prior to dissection
Suppl. 5a spoki w; UAS-spok RNAi #4/+; tubG4, tubG80ts/+
spoki + brki Trip w; UAS-spok RNAi #4/+; tubG4, tubG80ts/UAS-brk RNAi Trip
Suppl. 5b spoki w; UAS-spok RNAi/+; tubG4, tubG80ts/UAS-spok RNAi
spoki + armS10 w; UAS-spok RNAi/UAS-armS10; tubG4, tubG80ts/UAS-spok RNAi
Suppl. 5c WT w1118
spoki w; UAS-spok RNAi/+; tubG4, tubG80ts/UAS-spok RNAi
Suppl. 5d-f spoki w; UAS-spok RNAi/+; tubG4, tubG80ts/UAS-spok RNAi
spoki + wtsi w; UAS-spok RNAi, UAS-wts RNAi/+; tubG4, tubG80ts/UAS-spok RNAi
Suppl. 6 spoki w; UAS-spok RNAi/+; tubG4, tubG80ts/UAS-spok RNAi
Suppl. 6a spoki + TSC2i w; UAS-spok RNAi, UAS-TSC2 RNAi/+; tubG4, tubG80ts/UAS-spok RNAi
Suppl. 6b tub>S6Ki w; UAS-S6K RNAi/+; tubG4, tubG80ts/+ kept at 29°C
Suppl. 6c-d ctrl wL3 w1118
Suppl. 7a spoki w; UAS-spok RNAi/+; tubG4, tubG80ts/UAS-spok RNAi
spoki + TSC2i (30B + 40D) w; UAS-spok RNAi, UAS-TSC2 RNAi/+; tubG4, tubG80ts/UAS-spok RNAi
Suppl. 7b spoki w; UAS-spok RNAi #4/+; tubG4, tubG80ts/+
spoki + TSC2i (30B) w; UAS-spok RNAi #4, UAS-TSC2 RNAi (30B)/+; tubG4, tubG80ts/+
Suppl. 7c spoki w; UAS-spok RNAi #4/+; tubG4, tubG80ts/+
spoki + TSC2i Trip w; UAS-spok RNAi #4/+; tubG4, tubG80ts/TSC2 RNAi Trip
Suppl. 8a -Ubx actin>FRT>CD2>FRT>Gal4/y; UAS-TSC2 RNAi, UAS-spok RNAi #4/+; phmG4, tubG80ts/+
+Ubx actin>FRT>CD2>FRT>Gal4/UbxFlp; UAS-TSC2 RNAi, UAS-spok RNAi #4/+; phmG4, tubG80ts/+
Suppl. 8b phm>spoki + TSC2i w; UAS-spok RNAi, UAS-TSC2 RNAi/+; phmG4, tubG80ts/UAS-spok RNAi
Suppl. 8d spoki w; UAS-spok RNAi/+; tubG4, tubG80ts/UAS-spok RNAi
spoki + CycD + Cdk4 w; UAS-spok RNAi, UAS-CycD; UAS-spok RNAi, UAS-Cdk4/tubG4, tubG80ts
Suppl. 9a spoki w; UAS-spok RNAi/+; tubG4, tubG80ts/UAS-spok RNAi
Suppl. 9b spoki + TSC2i w; UAS-spok RNAi, UAS-TSC2 RNAi/+; tubG4, tubG80ts/UAS-spok RNAi
Suppl. 9c TubLexAts>LexAop-spoki + rnG4 +/- TSC2i tubLexA/+; UAS-TSC2 RNAi/+; lexAop-spok RNAi/rnG4,tubG80ts
Suppl. 9e wtsP2 + nub>brki w; nubG4/UAS-brk RNAi; wtsP2/wtsP2
Suppl. 9f wL3 w1118
Suppl. 9g unk-GFP unk-GFP
Suppl. 10a-c,e spoki w; UAS-spok RNAi/+; tubG4, tubG80ts/UAS-spok RNAi
Suppl. 10c tub>GSK3i w; UAS-S6K RNAi/+; tubG4, tubG80ts/+ kept at 29°C
Suppl. 10d hh>GFP, GSK3ß RNAi w; UAS-GFP/UAS-GSK3ß RNAi; hhG4/+
hh>GFP, Akt RNAi w; UAS-GFP/UAS-Akt RNAi; hhG4/+
Suppl. 10f spoki + Foxo-GFP w; UAS-spok RNAi/+; UAS-spok RNAi, UAS-Foxo-GFP/tubG4, tubG80ts
Suppl. 10g spoki + RagC S54N w; UAS-spok RNAi/+; UAS-spok RNAi, UAS-RagC S54N/tubG4, tubG80ts

Wild animals	This study did not involve wild animals.
Field-collected samples	This study did not involve samples collected from the field.
Ethics oversight	This study did not require an ethical approval.

Note that full information on the approval of the study protocol must also be provided in the manuscript.
